# Whole-Exome Sequencing in a Cohort of High Myopia Patients in Northwest China

**DOI:** 10.3389/fcell.2021.645501

**Published:** 2021-06-18

**Authors:** Yang Liu, Jin-Jin Zhang, Shun-Yu Piao, Ren-Juan Shen, Ya Ma, Zhong-Qi Xue, Wen Zhang, Juan Liu, Zi-Bing Jin, Wen-Juan Zhuang

**Affiliations:** ^1^School of Basic Medical Sciences, Third Clinical Medical College of Ningxia Medical University (People’s Hospital of Ningxia Hui Autonomous Region), Yinchuan, China; ^2^Clinical Medical College, Ningxia Medical University, Yinchuan, China; ^3^Ningxia Eye Hospital, People’s Hospital of Ningxia Hui Autonomous Region, Third Clinical Medical College of Ningxia Medical University, Yinchuan, China; ^4^Beijing Institute of Ophthalmology, Beijing Tongren Eye Center, Beijing Tongren Hospital, Capital Medical University, Beijing Ophthalmology and Visual Science Key Laboratory, Beijing, China; ^5^Department of Ophthalmology, Affiliated Hospital of Qingdao Binhai University, Qingdao, China

**Keywords:** high myopia, cohort, mutation, gene, Northwest China

## Abstract

High myopia (HM) is one of the leading causes of visual impairment worldwide. In order to expand the myopia gene spectrum in the Chinese population, we investigated genetic mutations in a cohort of 27 families with HM from Northwest China by using whole-exome sequencing (WES). Genetic variations were filtered using bioinformatics tools and cosegregation analysis. A total of 201 candidate mutations were detected, and 139 were cosegregated with the disease in the families. Multistep analysis revealed four missense variants in four unrelated families, including c.904C>T (p.R302C) in *CSMD1*, c.860G>A (p.R287H) in *PARP8*, c.G848A (p.G283D) in *ADAMTSL1*, and c.686A>G (p.H229R) in *FNDC3B*. These mutations were rare or absent in the Exome Aggregation Consortium (ExAC), 1000 Genomes Project, and Genome Aggregation Database (gnomAD), indicating that they are new candidate disease-causing genes. Our findings not only expand the myopia gene spectrum but also provide reference information for further genetic study of heritable HM.

## Introduction

Myopia, also known as shortsightedness, is globally increasing in prevalence. Approximately 108 million people are currently affected, and myopia is expected to become the leading cause of blindness across the globe (Bourne et al., 2013). In East Asian countries, the prevalence of myopia has been reported to be twice as high as in western countries (Pan et al., 2015).

High myopia (HM), which is the extreme form of myopia, is diagnosed in cases with refractive error worse than or equal to −6 diopters (D) and/or ocular axial length (AL) >26.00 mm (Percival, 1987; Young et al., 2007). Recently meta-analyses have estimated that the prevalence of myopia will rise to 49.8% in 2050 and that the prevalence of HM will increase to 9.8% (Holden et al., 2016). The complications associated with HM, such as cataract, glaucoma, retinal detachment, chorioretinal degeneration, and choroidal neovascularization, may lead to severe visual impairment and blindness (Morgan et al., 2012; Tham et al., 2018).

Both environmental and genetic factors are known to contribute to HM (Morgan et al., 2012).

Familial aggregation and twin studies have demonstrated that genetic factors play critical roles in the development of HM (Katz et al., 1997; Hammond et al., 2001). To date, 25 loci (MYP1–MYP3, MYP5–MYP26) associated with HM have been discovered *via* genome-wide association studies (GWAS), family-based linkage analyses, and whole-exome sequencing (WES) and documented in the Online Mendelian Inheritance in Man (OMIM) (Zhang, 2015; Fan et al., 2016; Cai et al., 2019a). However, only a limited number of causative genes have been identified (Cai et al., 2019a). WES has identified several genes responsible for HM, including two X-linked genes, *OPN1LW* (Li et al., 2015) and *ARR3* (Xiao et al., 2016); six autosomal dominant genes, *ZNF644* (Shi et al., 2011a), *SCO2* (Tran-Viet et al., 2013), *SLC39A5* (Guo et al., 2014), *P4HA2* (Guo et al., 2015), *BSG* (Jin et al., 2017), and *CCDC111* (Zhao et al., 2013); and three autosomal recessive genes, *LEPREL1* (Mordechai et al., 2011), *LRPAP1* (Aldahmesh et al., 2013), and *CTSH* (Jiang et al., 2015). Nevertheless, these genes explain the pathogenesis of HM in a limited number of patients (Kloss et al., 2017). Therefore, numerous HM-related genes surely remain to be discovered. In this study, we identified candidate mutations and genes in 27 non-syndromic families with HM from Northwest China.

## Materials and Methods

### Patient Subjects

Twenty-seven families with HM were recruited in this study. The study was performed in accordance with the tenets of the Declaration of Helsinki. Written informed consent was obtained from the participants or their statutory guardians, with approval from the institutional review board of the People’s Hospital of Ningxia Hui Autonomous Region, Third Clinical Medical College of Ningxia Medical University. Refractive error and AL of the patients in each family were concisely recorded in Table 1, a collection of the upper and lower limits of clinical data of all patients in each of the 27 families. The pedigree and clinical information of the 27 families were integrated in Supplementary Figure 1. Genomic DNAs were extracted from peripheral blood samples.

**TABLE 1:** Clinical data from 27 families with high myopia.

Family	Proband age	Affected individuals	SE range	SE median	AL range	AL median
85	57	2	−19.50/−23.75	−20.25	29.61/31.72	30.85
89	19	1	−9.75/−10.11	−9.93	27.80/27.90	27.85
90	19	1	−7.75/−8.25	−8.01	28.17/28.35	28.26
91	19	2	−8.50/−16.50	−11.88	27.52/28.67	27.81
92	23	1	−7.75/−8.75	−8.25	26.85/26.87	26.86
93	21	2	−10.00/−11.75	−10.81	24.75/27.22	25.85
94	22	2	−8.00/−9.25	−8.50	27.01/27.53	27.32
95	21	1	−6.75/−7.25	−7.00	26.45/26.75	26.60
96	18	1	−6.75/−7.00	−6.86	26.65/26.71	26.68
97	23	2	−6.25/−14.00	−11.25	26.58/29.86	28.03
99	19	1	−8.75/−9.00	−8.88	27.97/28.18	28.08
100	22	2	−6.00/−7.00	−6.50	24.98/26.27	25.60
101	20	1	−8.50/−8.75	−8.63	27.32/27.37	27.35
102	17	1	−7.50/−7.50	−7.50	27.46/27.50	27.48
103	18	1	−8.00/−8.75	−8.38	26.96/27.28	27.12
104	15	2	−5.75/−10.50	−6.71	26.12/26.91	26.28
105	33	1	−8.25/−9.25	−8.75	27.43/27.63	27.53
106	27	2	−5.50/−11.50	−8.56	26.05/27.33	26.62
107	20	3	−6.50/−13.00	−9.63	25.10/27.33	25.85
109	19	1	−7.25/−8.25	−7.75	27.14/27.75	27.45
110	24	1	−7.75/−9.25	−8.50	26.51/26.79	26.65
111	18	1	−6.00/−6.00	−6.00	26.65/26.76	26.71
112	23	1	−10.00/−10.50	−10.25	27.35/27.36	27.36
113	21	2	−12.75/−14.50	−9.31	26.64/26.91	26.42
114	18	1	−7.75/−9.00	−8.38	26.85/27.16	27.01
115	22	2	−6.00/−9.75	−8.28	26.60/28.55	27.62
116	20	1	−10.00/−10.50	−10.25	27.96/28.17	28.07

*SE, refractive error; AL, axial length.*

All 27 families included in this study were from Ningxia Hui Autonomous Region.

Patients were recruited according to the following inclusion criteria: (1) refractive error worse than or equal to −6 D and/or AL >26.00 mm; and (2) no other known ocular diseases or systemic disorders.

### Whole-Exome Sequencing

Whole-exome sequencing was performed as previously described with the Exome Enrichment V5 Kit (Agilent Technologies, United States) (Jin et al., 2018; Cai et al., 2019b; Zhou et al., 2020). DNA fragments were sequenced on Illumina HiSeq 2000 Analyzers (90 cycles per read). The Illumina libraries were prepared and generated using a sequencing platform (Hiseq2000; Illumina, Inc.), according to the manufacturer’s instructions. Local realignments, quality control text, and variant calling were assembled using the Genome Analysis Toolkit (GATK). All sequencing reads were mapped against a human reference genome (hg19/GRCH37) with BWA-MEM software as described (Kumar et al., 2009). WES yielded a mean read depth of ∼30×. Median coverage of the targeted regions was >95%.

### Variant Filtering

We focused on rare variants in ocular diseases and HM; the ocular disease genes were assembled from the databases: genes associated with the myopia phenotype term (HP:0000545) in Human Phenotype Ontology (HPO^1^) or ocular disease genes from RetNet database^2^ and OMIM^3^. The complete list of genes associated with myopia and ocular disease was shown in Supplementary Table 1.

**FIGURE 1:**
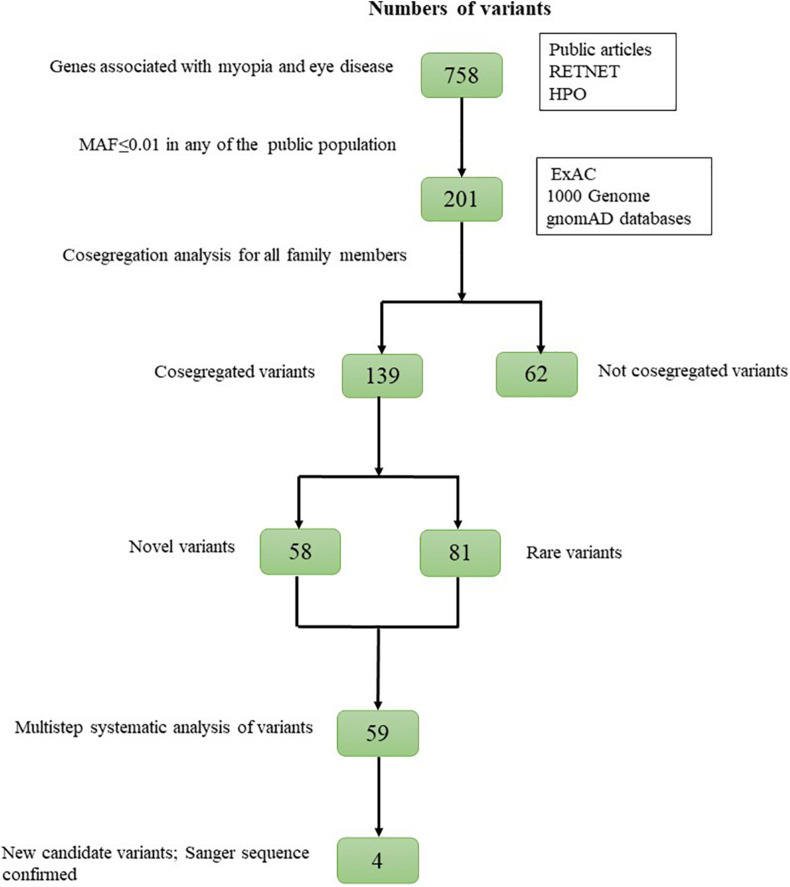
Overview of the variant filtering strategy.

The WES results from patients with HM were filtered as follows: (1) variants outside of the exonic splicing site predicted by the Berkeley Drosophila Genome Project^4^ were excluded; (2) synonymous variants were excluded without altering splice-site regions; (3) variants with minor allele frequencies (MAFs) greater than or equal to 0.01 in any of the three public population databases [Exome Aggregation Consortium (ExAC), 1000 Genomes Project (1000G), and Genome Aggregation Database (gnomAD)] were excluded; (4) variants that were not heterozygous in all AD families were excluded, as the variants that were not homozygous in all AR families. Meanwhile, *de novo* variants were also excluded, and these *de novo* variants were displayed in Supplementary Table 2. After these filtering steps, cosegregation analysis was performed for all family members. Finally, variant scores corresponding to the quality of biological and statistical evidence were assigned. Strong candidates were validated by Sanger sequencing (Figure 1).

All identified variants were assessed with the following tools and databases. The pathogenicity of gene mutations was predicted with 10 bioinformatics tools:

SIFT (Adzhubei et al., 2010)^5^, PolyPhen-2-HDIV, PolyPhen-2-HVAR (Schwarz et al., 2010)^6^, Mutation Taster (Desmet et al., 2009)^7^, LRT, Mutation Assessor, FATHMM, Radial SVM, LR, and DANN. Splicing mutations were analyzed with Human Splicing Finder software (Huang et al., 2015)^8^. Mutations with a MAF were evaluated with the ExAC^9^, 1000G (Bahcall, 2015) (1000G^10^), and gnomAD^11^. The RaptorX structure prediction web server^12^ was used to simulate the wild-type and mutant protein three-dimensional (3D) models. Changes in protein folding and crystal structure were visualized with PyMol software (version 1.5).

## Results

### Strong Candidate Genetic Causes Yielded by Computational Assessments and Cosegregation Analysis

**FIGURE 2:**
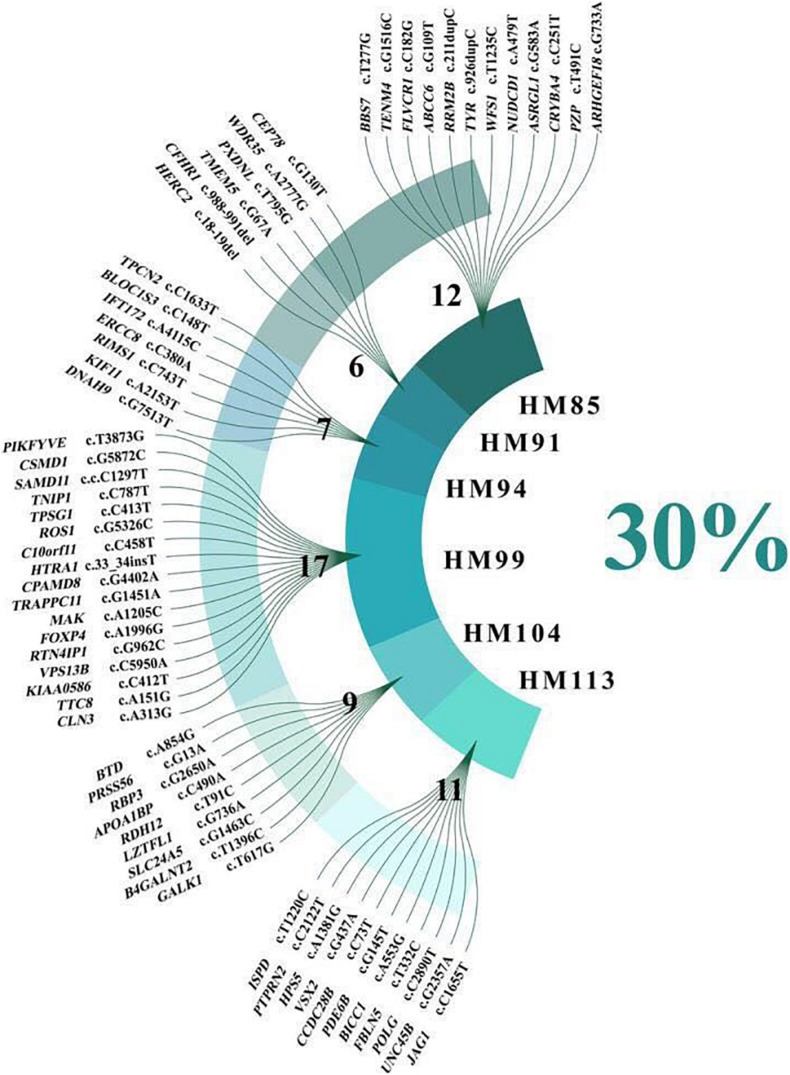
Sixty-two variants related to high myopia and ocular disease that were not cosegregated with high myopia.

A total of 201 potential variants were identified in 27 families: 139 were well cosegregated with the disease in 15 families. Sixty-two variants not cosegregated were excluded (Figure 2). Each variant was reported at a frequency of less than 1 in 100 alleles in the ExAC, 1000G, and gnomAD. For the 139 genes that cosegregated, none of the variants were identified in unaffected family members (refractive error less than 2.00 D). Fifty-eight of the 139 variants were novel changes that were unreported in public databases (Supplementary Table 3). Eighty-one of the 139 variants were rare changes (less than 1 in 1,000 alleles in any population) (Supplementary Table 4). Among 139 variants, 59 had been presented in 10 pathogenicity prediction tools with biological relevance (Supplementary Figure 2).

**FIGURE 3:**
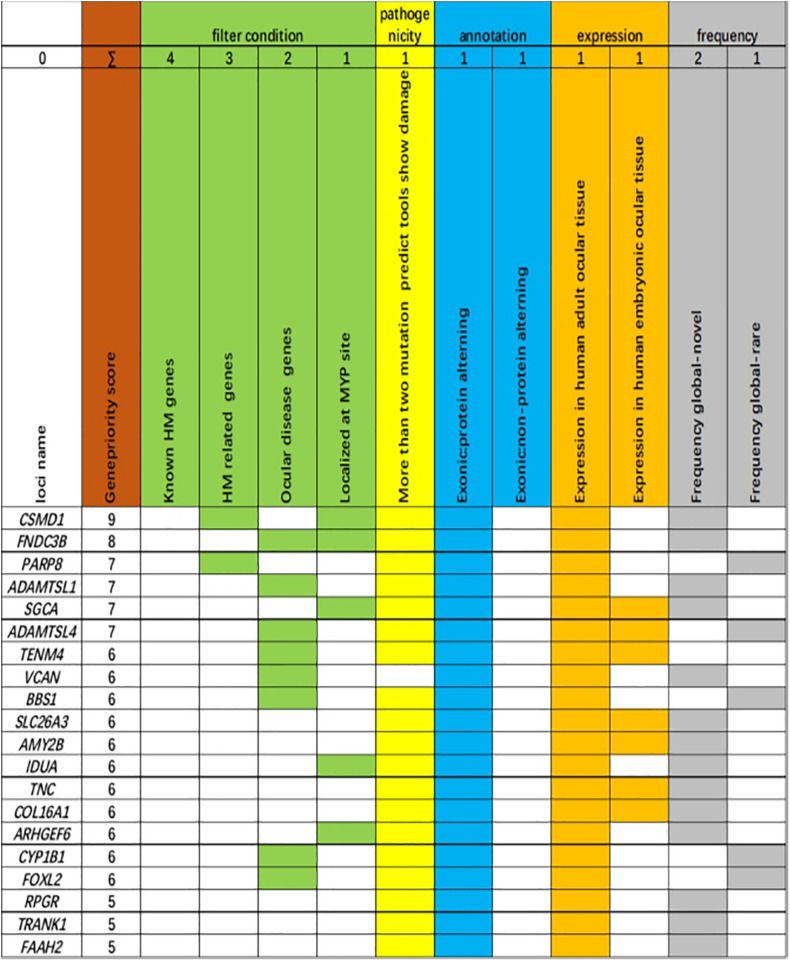
Genes ranked according to biological and statistical evidence. Genes were ranked based on 11 categories that can be divided into five categories: filter condition [green: genes are related to known high myopia (HM) genes, HM-related genes, ocular disease genes, or localized at the MYP site], pathogenicity (light yellow: more than two mutation predict tools show damage), annotation (light blue: genetic variant harboring an exonic protein altering variant or non-protein-altering variant), expression (dark yellow: expression in adult human ocular tissue, expression in human embryonic ocular tissue), and frequency [light dark: frequency global in Exome Aggregation Consortium (ExAC) database, novel; frequency global in ExAC database, rare].

The 20 variants with the highest scores are shown (Figure 3). Four missense variants in the *CSMD1*, *PARP8*, *ADAMTSL1*, and *FNDC3B* genes in four unrelated families achieved the highest scores, including c.904C>T (p.R302C) in *CSMD1*, c.860G>A (p.R287H) in *PARP8*, c.G848A (p.G283D) in *ADAMTSL1*, and c.686A>G (p.H229R) in *FNDC3B*. These variants were well cosegregated with the disease. Further analysis was performed according to the guidelines of the American College of Medical Genetics and Genomics (Bahcall, 2015). After these multistep systematic analyses, these genetic variants were estimated as potentially pathogenic and were rare or absent from the ExAC, 1000G, and gnomAD. The scores of DANN in *CSMD1*, *PARP8*, *ADAMTSL1*, and *FNDC3B* were 0.999, 0.915, 0.998, and 0.98, respectively.

### Candidate Genes Identified

**FIGURE 4:**
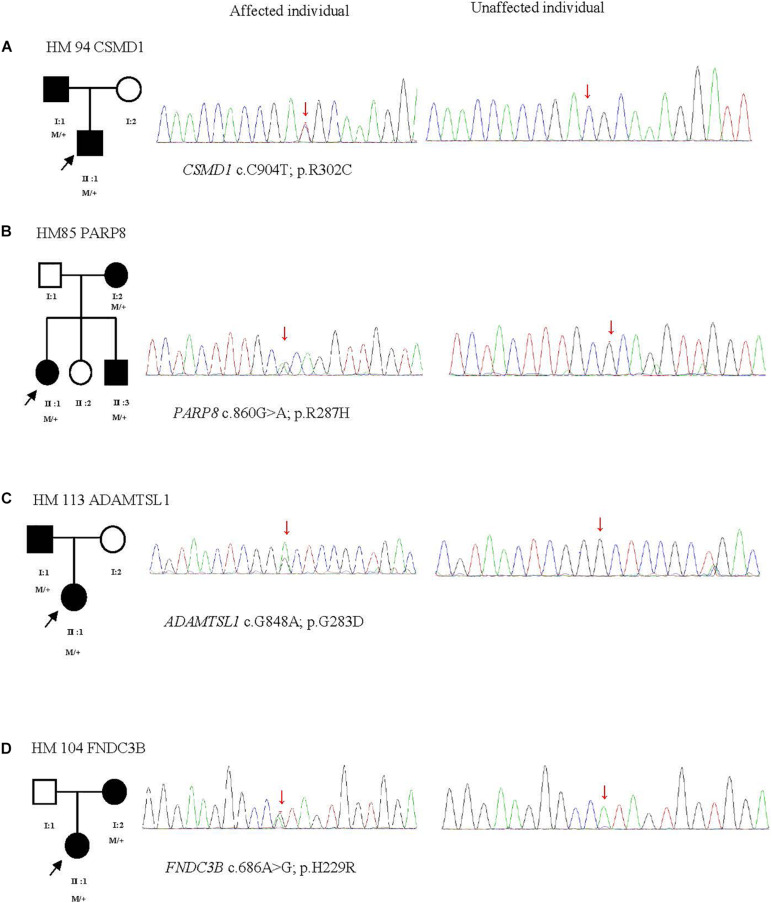
Four potentially pathogenic variants detected in this study. The black arrow represents the patient. +, wild-type; M, mutation. From left to right: pedigree plots of mutations, sequences from affected individual with identified mutation, sequences from unaffected control **(A–D)**.

**FIGURE 5:**
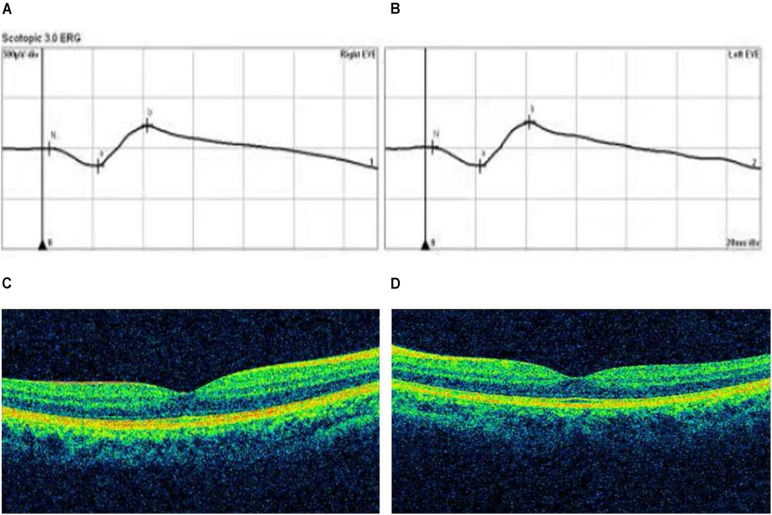
Optical coherence tomography (OCT) and electroretinogram (ERG) results of proband in Family 94. **(A,B)** Standard scotopic response of full-field ERG of the right and left eye showing normal b-wave amplitude. **(C,D)** OCT images of the right and left eye reveal the normal retinal appearance.

**FIGURE 6:**
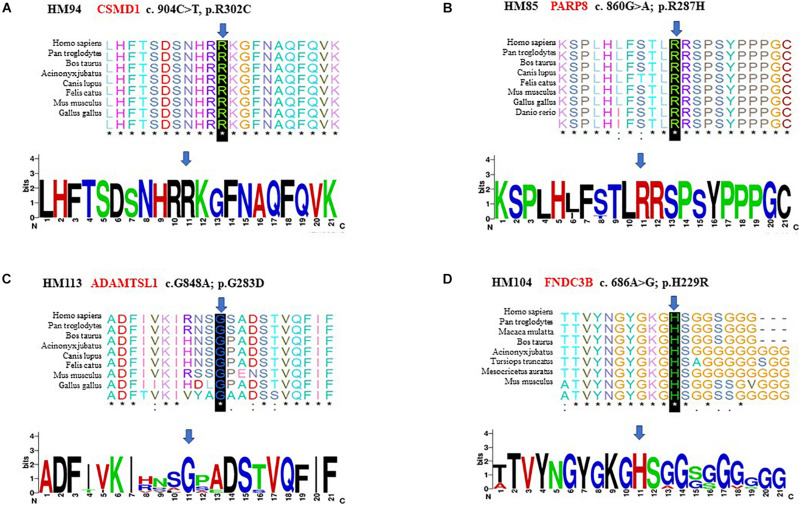
Conservation analysis revealed evolutionary conservation of the mutation. It shows multiple alignments of the amino acids from different species. The arrow indicates the location of the mutation **(A–D)**.

**FIGURE 7:**
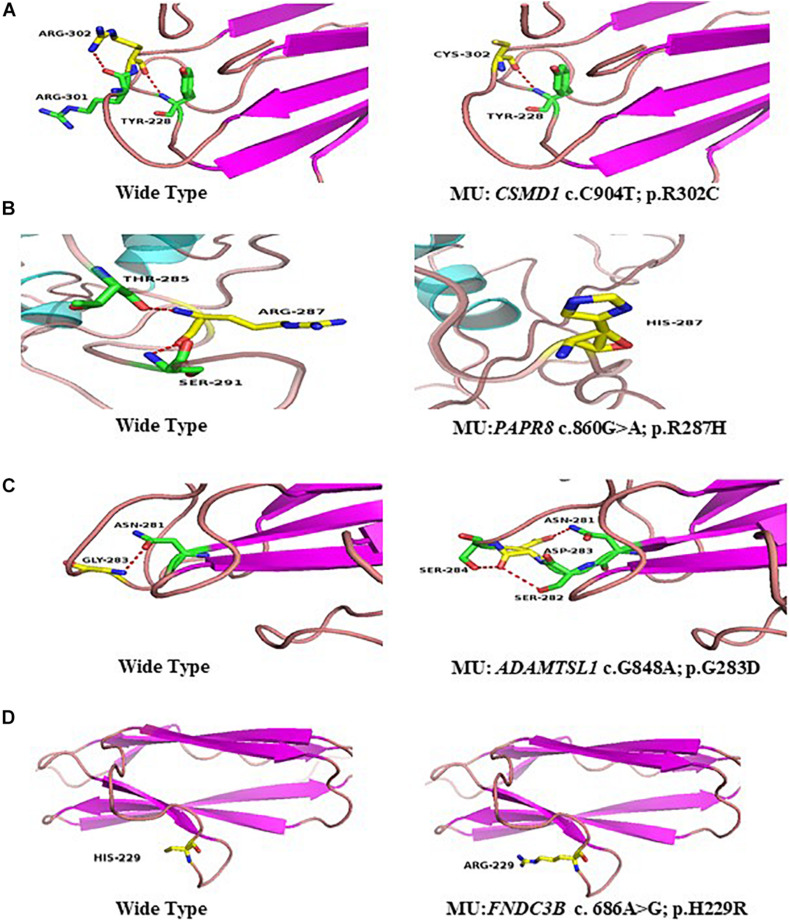
Predicted three-dimensional (3D) structure of proteins. Predicted crystal structures of wild-type (left) and mutant (right) proteins. Yellow shows residue of wild-type and mutant; green represents residues interact with wild-type (left), and mutant residue (right) **(A–D)**.

The p.R302C substitution (c.904C>T) in *CSMD1* was detected in a 22-year-old man and his father (Figure 4A). The individual and his father had refractive error less than −8.50 D in both eyes, with AL >27 mm, without oculopathy or systemic diseases. Interestingly, proband and their affected family member both had HM before school age according to their self-report. Electroretinogram (ERG) and optical coherence tomography (OCT) examinations revealed the normal retinal appearance of proband and his father (Figure 5 and Supplementary Figure 3), as did his mother in family 94 (Supplementary Figure 4). This mutation was predicted to be pathogenic by eight pathogenicity prediction tools (SIFT, Polyphen-2, Mutation Taster, Mutation Assessor, FATHMM, RadialSVM, LR, and DANN) and listed as novel in the ExAC, 1000G, and gnomAD. The substitution p.R302C occurred at a highly conserved region in multiple orthologous sequence alignments, which demonstrates that the associated protein plays an important role (Figure 6A), and this site may have an important effect on protein function. Three-dimensional modeling demonstrated the absence of a bond between the mutated cysteine at residue 302 and the arginine at residue 301 (Figure 7A).

A heterozygous mutation in *PARP8* was detected in a 25-year-old woman and her mother (Figure 4B), both of whom had extreme myopia, with average spherical equivalent refractive error greater than −20 D and AL >29 mm. This mutation was also detected in the proband’s younger brother, who had AL of 25.06 mm in the right eye and 25.14 mm in the left eye. But there were no other symptoms of oculopathy or systemic diseases. The c.860G>A mutation is a rare single-nucleotide polymorphism (SNP) (rs142224685) located in exon 11. The pathogenicity of the p.R287H mutation in *PARP8* (c.860G>A) was verified by SIFT, Mutation Taster, and PolyPhen-2. Furthermore, the p.R287H variant was detected as an SNP with a MAF of 0.0014 in ExAC. This amino acid mutation is evolutionarily conserved among species (Figure 6B). Three-dimensional protein modeling demonstrated the absence of a bond between mutated residue 287 and residues 285 and 291 (Figure 7B).

The c.848G>A, p.G283D variant in *ADAMTSL1* was identified in a 21-year-old female (Figure 4C), whose spherical equivalent refractive error was −15.25 D in the right eye, with an AL of 26.64 mm and −13.50 D in the left eye with AL 26.91 mm. This mutation in *ADAMTSL1* was a novel conservative mutation (Figure 6C) that was predicted to be disease-causing by SIFT, Polphen-2, and Mutation Taster. Three-dimensional structural modeling revealed a newly formed bond between residue 283 and residues 282 and 284 (Figure 7C), and this mutation is absent in the ExAC, 1000G, and gnomAD, indicating its disease causality.

A heterozygous mutation (c.686A>G; p.H229R) in *FNDC3B* was found in a proband from Family 104 and her mother (Figure 4D). The proband, a 15-year-old female, presented with concomitant severe anisometropia. Her spherical equivalent refractive error was −6.25 D in the right eye and −10.50 D in the left eye. According to the results of the corneal topography, the thickness of the thinnest point is 504 μm, and ^*K*^m is 56.2 D for her left eye (Supplementary Figure 5). The p.H229R mutation in *FNDC3B* (c.686A>G) was predicted to be pathogenic by six *in silico* tools (SIFT, Polphen-2, Mutation Taster, LRT, Mutation Assessor, and DANN). The mutation was in a region extremely conserved across eight homologous species (Figure 6D), in line with the changes of 3D protein folding (Figure 7D).

### Mutations in Known Genes Responsible for High Myopia

The screening of 11 known HM genes revealed four variants to be highly pathogenic, including c.518A>T (p.D173V) in *SCO2*, c.3266A>G (p.Y1089C) in *ZNF644*, c.758C>A (p. A253E) in *SLC39A5* (Family 89), and c.577G>A (p. D193N) in *SLC39A5* (Family 95). Notably, Families 89 and 95 exhibited an AR mode of inheritance. We detected the same mutation in the mother of the proband from Family 89 and the father of the proband from Family 95. The c.758C>A mutation in *SLC39A5*, which was predicted to be deleterious by eight of the 10 computational tools, was absent from the 1000G, ExAC, and gnomAD East Asian databases. The c.577G>A mutation in *SLC39A5*, which was predicted to be deleterious by Polyphen2_HDIV and MutationTaster, was extremely rare in healthy populations. The 1000G and ExAC databases listed MAF as 0.001 and 0.00007462 and MAF in gnomAD is 0.00008761, well below the Popmax Filtering AF 0.0001781 in the East Asian population.

*SCO2*, located at MYP6, was identified as an AD gene. The c.518A>T (p.D173V) mutation in *SCO2* was detected in Family 111, with a recessive mode of inheritance.

This variant was listed as novel in 1000G and gnomAD, associated with (MAF) 0.0000269 in the ExAC database. The SIFT and FATHMM predicted that this mutation would be deleterious. The MAF of c.3266A>G (p.Y1089C) in *ZNF644*, identified in the proband and his father of Family 115, was 0.001in 1000G, 0.0003 in the ExAC, and 0.002456 in gnomAD. This mutation was predicted to be deleterious by five of 10 computational tools.

## Discussion

Up to now, only a few genes responsible for non-syndromic myopia have been discovered. In our previous study of large-scale screening of eight causative genes in 731 patients with HM, we merely identified mutations in 6.16% cases (Cai et al., 2019c). In this study, we identified four new candidate genes associated with HM. All mutations in these genes affect the function of the coding region according to the 3D structure model, and *CSMD1*, *ADAMTSL1*, and *FNDC3B* were absent in three examined databases (ExAC, 1000G, gnomAD). SIFT, PolyPhen-2-HDIV, PolyPhen-2-HVAR, Mutation Taster, LRT, Mutation Assessor, FATHMM, RadialSVM, and LR were employed to assess the mutation changes in protein function and mutation changes in protein spatial conformation, which further cause harmful changes in physiological functions (Mahfuz and Khan, 2020).

*CSMD1*, located on MYP10 and mapped to chromosome 8p23.2, was highly expressed in the central nervous system and epithelial tissues. Furthermore, Wan et al. (2018) found that the *CSMD1* gene is expressed at extremely high levels in peripheral retina and the area surrounding the macula. It seems that *CSMD1* plays a critical role in the growth of cones, including signal transduction and matrix adhesion (Kraus et al., 2006). The *de novo* mutations of *CSMD1* have been reported in a Chinese family with early-onset high myopia (EOHM) (Jin et al., 2017). We hypothesize *CSMD1* is also a candidate gene for HM in addition to neurological effects. In our study, a heterozygous missense mutation (c.904C>T) in *CSMD1* was found in two patients of Family 94, which shows the characteristics of dominant inheritance and is consistent with family cosegregation. However, there were no significant retina changes from the OCT and ERG results of the proband and his father. Further molecular mechanism study about how *CSMD1* plays a role in the development of HM is needed. Meanwhile, our results provide more genetic evidence for the hypothesis that *CSMD1* is the causative gene for HM.

*PARP8* is a critical regulator of eukaryotic physiology, localized to the nuclear envelope, and resulted in cell morphology defects that showed potential functions for *PARPs* in the assembly or maintenance of membranous organelles (Vyas et al., 2013). As reported, *PARP* is the major risk factor associated with age-related cataract in central India (Ughade et al., 1998). Fan et al. (2012) first investigated the SNPs rs282544, rs2404958, rs32396, rs12055210, and rs11954386 on *PARP8*, which is closely related to the AL with p-values < 1 × 10^–5^.

Dongyan (2019) subsequently identified SNPs in *PARP8* gene including rs1195438, rs2404958, rs282544, and rs32396, which are significantly associated with HM in the Han southwest Chinese population. In this study, we also found a rare SNP (rs142224685) as a heterozygous missense mutation c.860G>A in *PARP8*. The research on the association between SNP on *PARP8* and HM needs to be further explored.

A recent study reported a heterozygous c.124T>C (p. Trp42Arg) mutation in the first thrombospondin type 1 (TSR) motif of *ADAMTSL1*, which is responsible for a complex disorder with features including developmental glaucoma, myopia, and/or retinal defects (Hendee et al., 2017). Moreover, *ADAMTSL1* caused the dislocation of microspherophakic lens that causes severe myopia, glaucoma, or cataract. Two modes of inheritance have been reported: autosomal dominant and autosomal recessive (Delhon et al., 2016).

The c.848G>A (p.G283D) variant in *ADAMTSL1*, identified in the proband and her father in Family 113 accompanied by extremely HM, shows a dominant inheritance model. This result is consistent with the previous study in that *ADAMTSL1* accounts for the myopia, especially for severe HM (Hendee et al., 2017).

*FNDC3B*, located on MYP8, which maps to chromosome 3q26.31, plays an important role in central corneal thickness (CCT), intraocular pressure, and anterior chamber angle depth (Rong et al., 2017). As we know, previous studies mainly focused on the association of *FNDC3B* with glaucoma and keratoconus (Shi et al., 2011b). CCT as one of the most heritable human traits was related to many eye diseases, especially for myopia (Pedersen et al., 2005). CCT reduction related to myopia was also reported (Hao et al., 2015; Rong et al., 2017). In our study, we identified a heterozygous mutation (c.686A>G; p.H229R) in *FNDC3B* from Family 104, and the left eye of the proband with higher refractive error showed typical keratoconus symptoms (−6.25 D/−10.50 D). Based on our findings, we speculate that the clinical phenotype of mutations in *FNDC3B* may be HM for some people, and it may also be keratoconus for other people, so whether it is an HM candidate disease-causing gene remains to be further evaluated.

The association of known HM genes with HM has been previously investigated in several studies (Jiang et al., 2015; Rong et al., 2017). For this study, we examined the associations within our own HM cohort. The screening of 11 known HM genes revealed four variants in *SCO2*, *ZNF644*, and *SLC39A5* to be highly potentially pathogenic.

*SCO2* was found to be related to HM in a large three-generation family in Europe, with nine affected individuals with HM (average spherical refractive error of −22.00 D). Four heterozygous mutations, p.G53^∗^, p.A114H, p.G140L, and p.A259V, in *SCO2* were identified; these mutations may truncate or destabilize the protein structure and lead to retinal neuronal thinning (Tran-Viet et al., 2013). Moreover, R120W, R112W, and A97V were reported in subsequent studies (Jiang et al., 2015; Wakazono et al., 2016).Tran-Viet et al. (2013) observed Sco2 protein localization in the retina, retinal pigment epithelium (RPE), and scleral wall in mouse ocular tissues. We further identified mutations A201P and I221V in *SCO2* in 2019 (Cai et al., 2019c). Here, a new variant c.518A>T (p.D173V) in *SCO2* is detected.

*ZNF644*, which is located at 1p22.2, can inhibit histone methyltransferase complex through G9a/GLP, functioning as a regulator of histone methyltransferase complex (Bian et al., 2015). *ZNF644* is widely expressed in eye, placenta, and liver and is assumed to play a pivotal role in changes in ocular wall. Mutations in this gene have been reported in previous studies to be associated with HM (Shi et al., 2011a; Jin et al., 2017). In this study, we detected a missense mutation c.3266A>G (p.Y1089C) in *ZNF644* from the proband and his father. Interestingly, while the son had HM, the father had normal vision. It seems that this situation does not correspond to the cosegregation of the clinical phenotype. Further functional studies are needed to elucidate this.

*SLC39A5*, located at 12q13.3, encodes zinc transporter (Xia et al., 2020). A heterozygous truncation mutation in the *SLC39A5* gene p.Y47^∗^ was first reported in 2014 (Guo et al., 2014). Subsequently, the missense mutation p.M304T was detected in the same family; p.G413A (Jiang et al., 2015), p.A84T, p.P87L, p.A319T, and p.A243fs^∗^140 were found to be responsible for HM (Feng et al., 2017; Liu et al., 2020). Other studies have shown that the bone morphogenetic protein (BMP)/transforming growth factor-beta (TGF-β) pathway is related to myopia, and c.141C>G, p.Y47^∗^ mutation may cause the loss of function (Jobling et al., 2009; Guo et al., 2014). It has been demonstrated that destruction of BMP/TGF-β pathway may lead to refractive errors (Wu et al., 2018). In the present study, two missense mutations, c.758C>A (p. A253E) and c.577G>A (p.D193N), in *SLC39A5* were detected in two unrelated families. The relationship between these mutations and functions needs to be further studied.

In our study, 62 variants that were highly potentially pathogenic and related to ocular disease or HM were incompatible with cosegregation; they may be used as references for subsequent research. Notably, our technology failed to detect variants in 12 families. On one hand, we set stringent screening criteria, which may have reduced the number of genes for selection. On the other hand, most published articles on HM genetics are focused on families with dominant patterns of inheritance, which means that families with dominant genetic patterns of inheritance may be easier to screen for genes associated with HM. In our study, 18 of 27 families had a recessively inherited clinical phenotype. HM is a very clinically heterogeneous disease, even between patients in the same family. Refractive status and AL are different between patients. This point was also verified in our investigation of the four families in the sibships (Families 85, 99, 100, and 107). In Family 85, the 14-year-old brother of the patient showed the eye AL above 25 mm, and his diopter was less than 3.00 D; with the increase in age, the patient’s brother was very likely to develop into HM characterized by excessive AL. The younger brother of the proband in Family 99 is now 13 years old with refractive error −1.75 D in the right eye and −2.25 D in the left eye. On the other hand, the AL of his eyes is about 25 mm. There is also a risk of developing axial HM. Interestingly, the situation of Family 107 is opposite to that of Families 85 and 99. The three sisters are patients, and all showed HM with high diopter and low AL.

Especially the two sisters of the proband showed the diopter was greater than −6.00 D with the AL less than 26 mm. The two patients in Family 100 are of similar age; the older sister is 22 years old and the younger brother is 21 years old. The refractive error and AL of their eyes are relatively similar, in addition to the common genetic factors. The consistency of their living background may also play an important role in the development of their myopia for HM is a disease in which environmental and genetic factors work together. Different life backgrounds, dietary habits, and near-work habits may all contribute to the development of HM.

This study had some limitations. Because environmental and genetic factors determine the manifestation of HM, environmental factors may be an important influencing factor that causes inconsistency between the genotype and the clinical phenotype. This study did not focus on the impact of environmental factors, which may explain our observations of non-cosegregated genes, and we did not carry out functional verification tests. However, analysis of the pathogenicity of gene mutations based on the interpretation standards and guidelines of gene mutations developed by the American College of Medical Genetics and Genomics (ACMG) showed that four new candidate genes met the evidence criteria for possibly pathogenic mutations. The mechanisms underlying the effects of these genetic variants in the context of HM require further elucidation through additional studies. In addition, we have not investigated the impact of copy number variation (Huang et al., 2017) and somatic mosaicism (Jin et al., 2007) in these patients. Finally, targeted exome sequencing including a panel of disease-causing genes, likewise the strategy of inherited retinal dystrophy (Huang et al., 2013, 2015; Xing et al., 2014; Chen et al., 2020), might be more appropriate for future genetic diagnosis of genetic HM.

## Conclusion

In conclusion, we identified 201 genetic variants related to the development of HM by WES and bioinformatics analysis. To our knowledge, this is the first study of WES in patients with HM from Northwest China. The findings presented four new candidate genes associated with HM, providing additional evidence of heritable HM in Chinese patients. These findings expand the mutation spectrum of myopia genes and provide clues for further genetic study of HM.

## Data Availability Statement

The datasets presented in this study can be found in online repositories. The names of the repository/repositories and accession number(s) can be found below: https://figshare.com/articles/dataset/Whole-exome_sequencing_in_a_cohort_of_high_myopia_patients_in_northwest_China/14480316, accession doi: 10.6084/m9.figshare.14480316.

## Ethics Statement

The studies involving human participants were reviewed and approved by the Institutional Review Board of People’s Hospital of Ningxia Hui Autonomous Region, Third Clinical Medical College of Ningxia Medical University. Written informed consent to participate in this study was provided by the participants’ legal guardian/next of kin.

## Author Contributions

W-JZ, Z-BJ, and JL designed and supervised the whole study. YL, J-JZ, and S-YP performed the experiments. YL, R-JS, YM, Z-QX, and WZ interpreted the results. YL drafted the manuscript. Z-BJ and W-JZ revised the manuscript. All authors contributed to the article and approved the submitted version.

## Conflict of Interest

The authors declare that the research was conducted in the absence of any commercial or financial relationships that could be construed as a potential conflict of interest.

## Footnotes

1 http://human-phenotype-ontology.github.io

2 http://www.sph.uth.tmc.edu/retnet/

3 https://www.omim.org/

4 http://www.fruitfly.org/

5 http://sift.jcvi.org/

6 http://genetics.bwh.harvard.edu/pph2/index.shtml

7 http://www.mutationtaster.org/

8 http://www.umd.be/HSF/

9 http://ExAC.broadinstitute.org/

10 ftp://1000genomes.ebi.ac.uk/vol1/ftp

11 https://gnomad.broadinstitute.org/

12 http://raptorx.uchicago.edu/
