# Supplementary material for: Whole-Exome Sequencing in a Cohort of High Myopia Patients in Northwest China
Source: Front Cell Dev Biol. 2021 Jun 18;9:645501. doi: 10.3389/fcell.2021.645501 (PMC8250434; doi:10.3389/fcell.2021.645501)
Supplement: Supplementary file 1 [file Data_Sheet_1.zip › Supplemental Figure 1.DOCX]

9 dominant families:


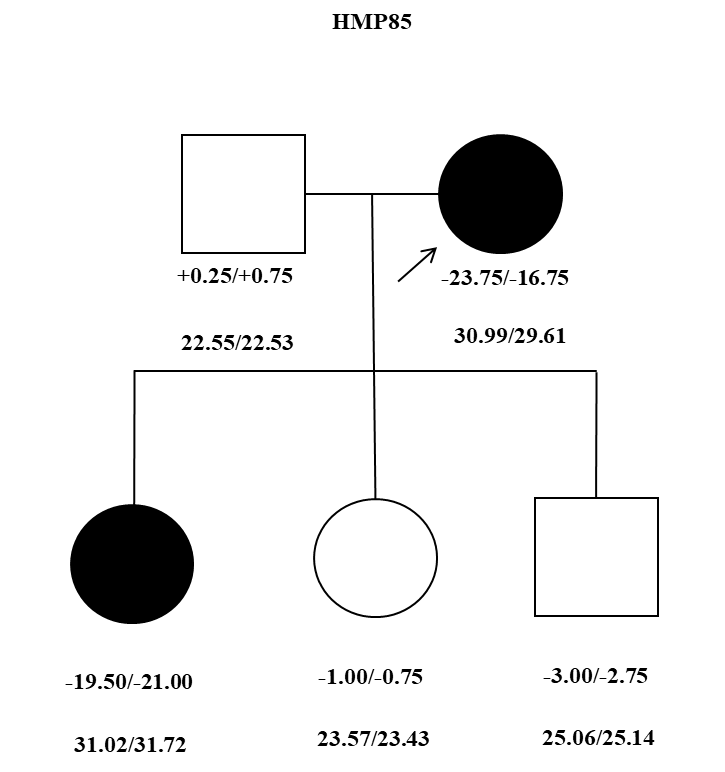


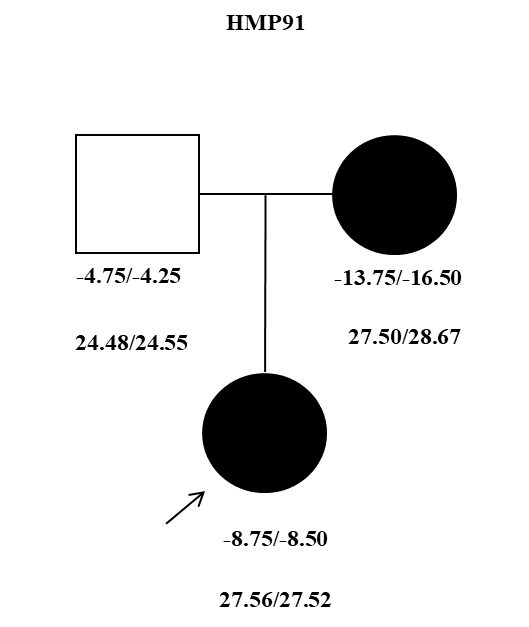


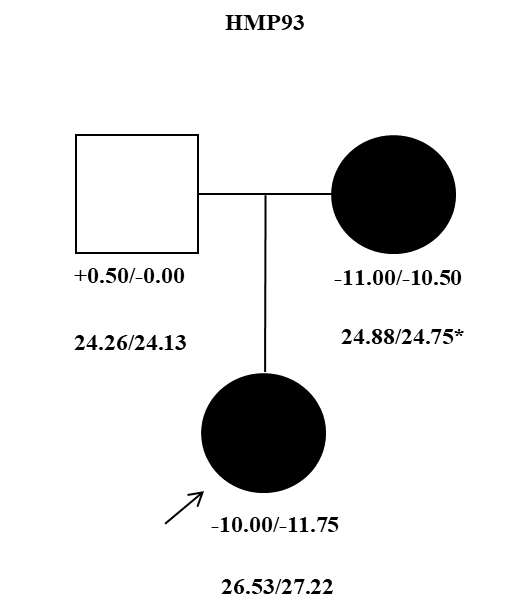


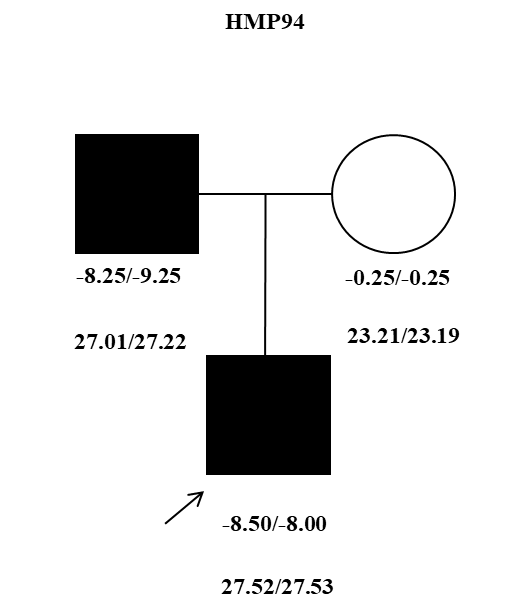


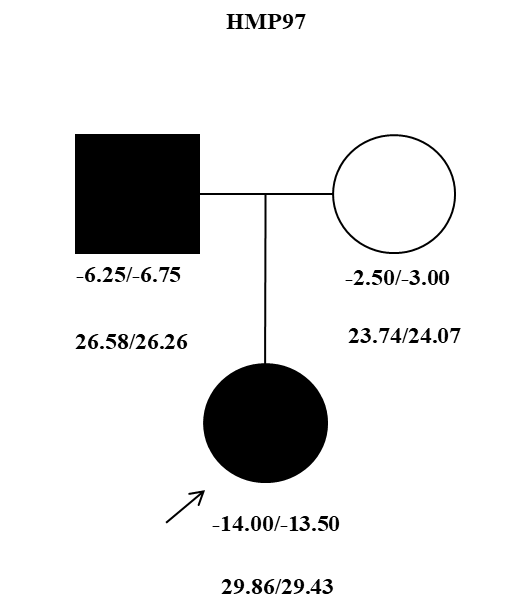


**26.47/26.64**

**26.12/26.91**

**25.79/25.74**

**HMP104**

**-6.25/-10.50**

**-5.75/-6.25**

**-5.75/-5.25**


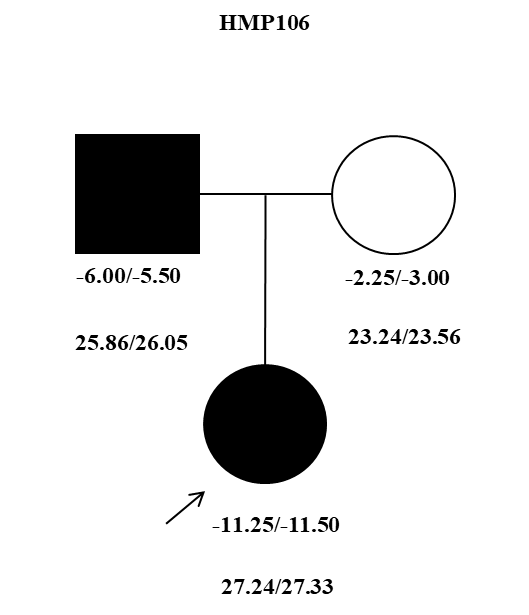


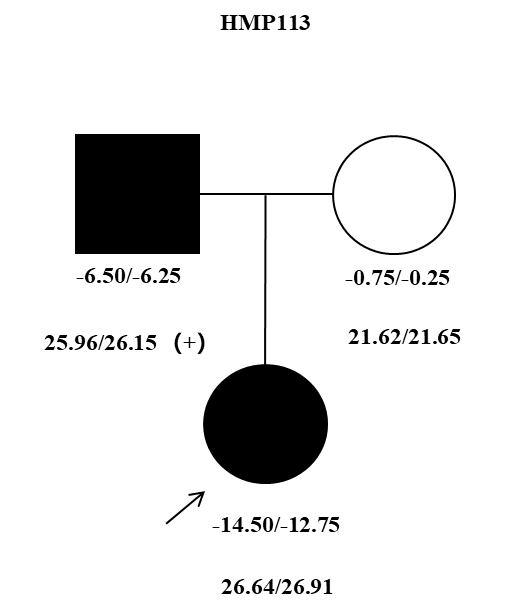


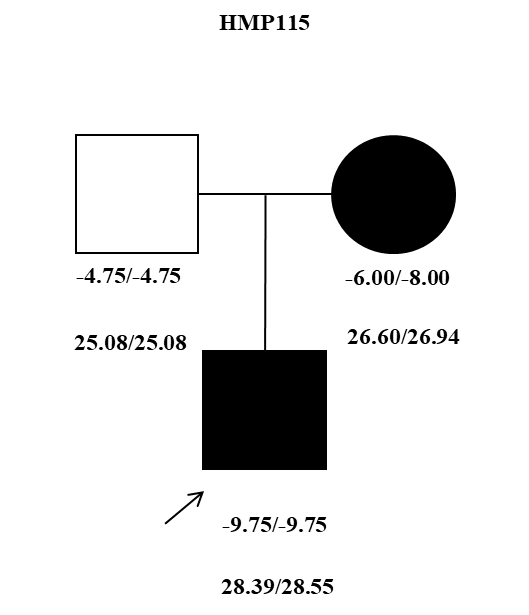


18 recessive families:


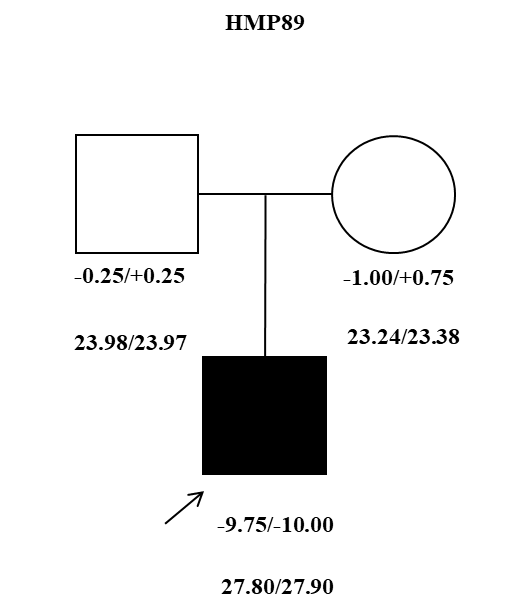


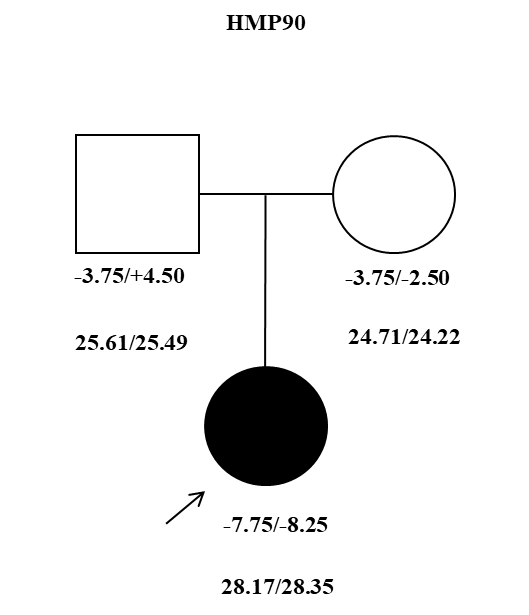


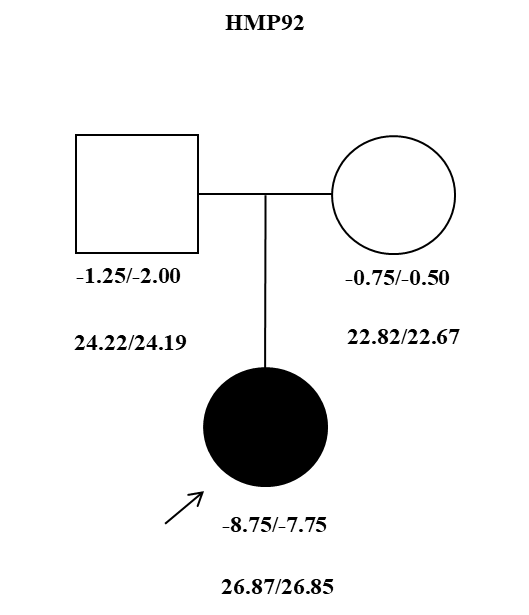


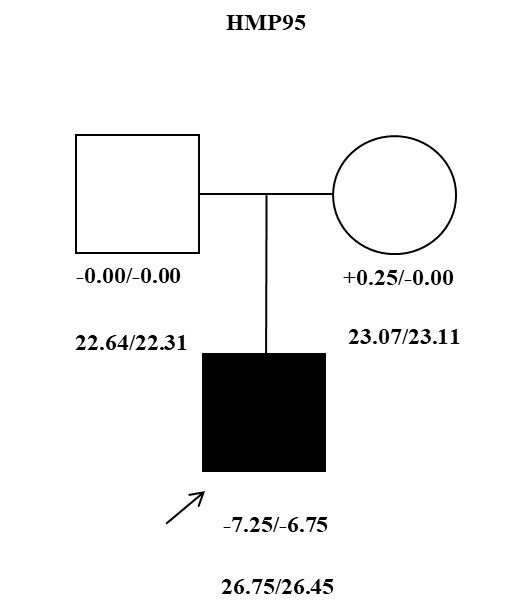


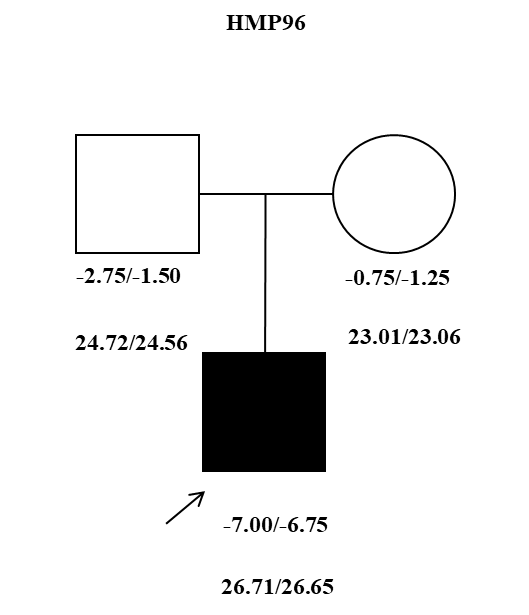


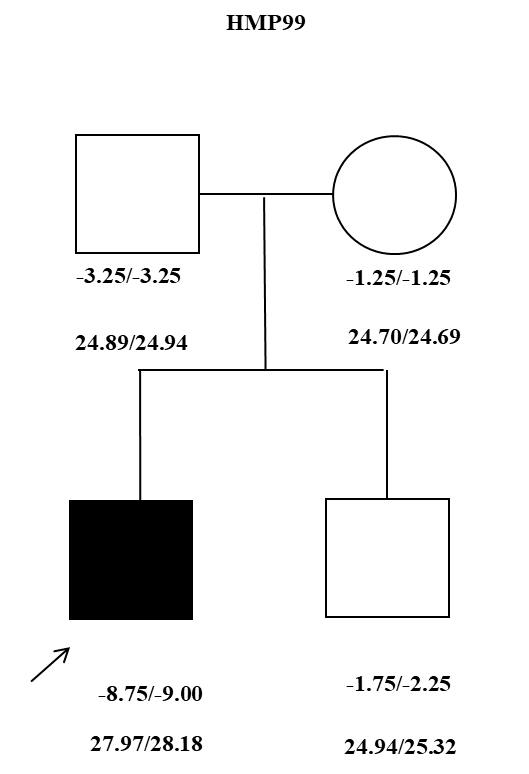


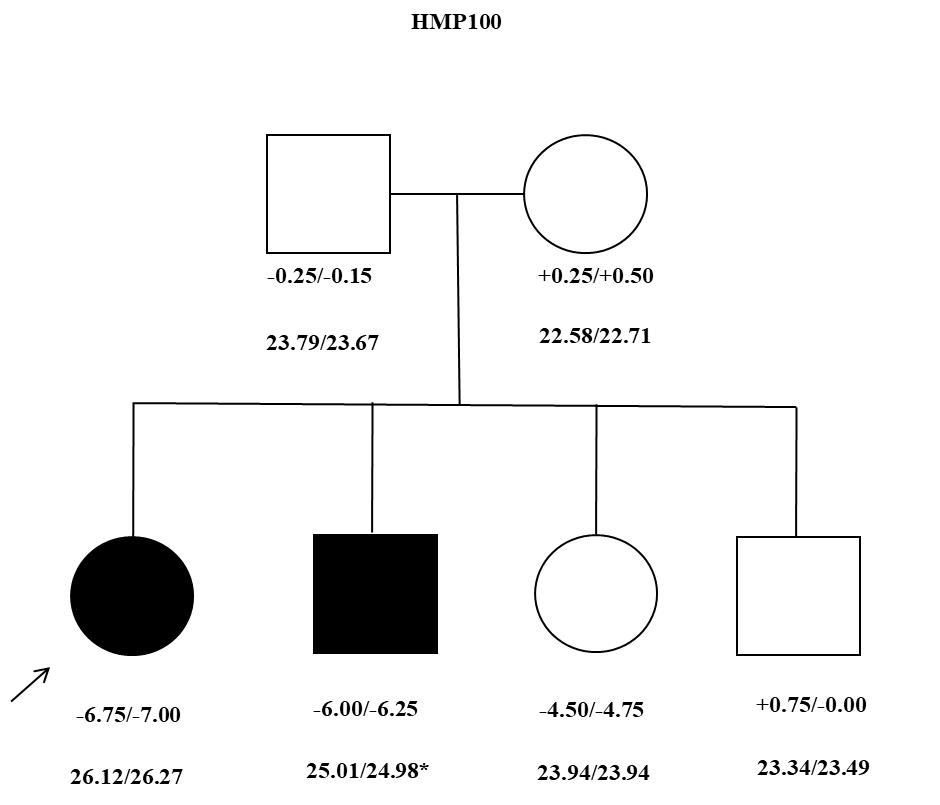


**23.96/23.85**

**27.32/27.37**

**24.63/23.91**

**HMP101**

**-8.50/-8.75**

**-2.50/-2.00**

**-4.75/-1.75**

**23.09/23.10**

**27.46/27.50**

**23.02/23.06**

**HMP102**

**-7.50/-7.50**

**-0.50/+0.75**

**+0.25/+0.50**

**24.05/24.19**

**27.28/26.96**

**23.97/23.10**

**HMP103**

**-8.75/-8.00**

**-5.25/-5.25**

**-0.50/+0.75**

**23.09/23.22**

**27.63/27.43**

**25.71/25.02**

**HMP105**

**-9.25/-8.25**

**+0.25/+0.50**

**-3.25/-1.75**

**23.23/23.35**

**22.19/22.29**

**HMP107**

**-0.50/-0.25**

**+0.25/-0.00**

**26.94/27.33**

**-12.25/-13.00**

**25.62/25.45**

**-4.75/-4.50**

**25.25/25.10***

**-7.00/-6.50**

**25.26/25.23***

**-9.25/-9.75**

**23.68/23.70**

**27.75/27.14**

**23.67/23.38**

**HMP109**

**-8.25/-7.25**

**-1.50/-1.00**

**-1.50/-1.25**

**22.78/22.76**

**26.79/26.51**

**23.35/23.08**

**HMP110**

**-9.25/-7.75**

**+1.00/+1.25**

**-2.00/-1.25**

**23.91/23.99**

**26.65/26.76**

**24.79/24.72**

**HMP111**

**-6.00/-6.00**

**-2.00/-1.75**

**+0.25/+0.25**

**23.44/23.34**

**27.36/27.35**

**24.54/24.70**

**HMP112**

**-10.00/-10.50**

**-0.50/-0.75**

**-0.25/-0.25**

**22.41/22.33**

**27.16/26.85**

**23.34/23.40**

**HMP114**

**-9.00/-7.75**

**-0.25/+0.25**

**-1.25/-1.25**

**22.78/22.67**

**27.96/28.17**

**22.75/22.91**

**HMP116**

**-10.00/-10.50**

**-1.00/-1.25**

**-0.25/-0.25**

**Supplemental Figure 1. pedigree and clinical information of 27 families.**

Black: affected; White: unaffected; The number upward represents the refractive error, and the number downward represents the length of the eye axis; *: The patient's refractive error is lower than -6.00D while the axial length of both eyes is less than 26mm; (+): The patient's refractive error is lower than -6.00D while the axial length of one eye is less than 26mm.
