# Supplementary material for: Whole-Exome Sequencing in a Cohort of High Myopia Patients in Northwest China
Source: Front Cell Dev Biol. 2021 Jun 18;9:645501. doi: 10.3389/fcell.2021.645501 (PMC8250434; doi:10.3389/fcell.2021.645501)
Supplement: Supplementary file 1 [file Data_Sheet_1.zip › Supplemental Figure 2.DOCX]

|  |  | **filter condition** | | | | **pathogenicity** | **annotation** | | **expression** | | **frequency** | |
| --- | --- | --- | --- | --- | --- | --- | --- | --- | --- | --- | --- | --- |
| **0** | **∑** | **4** | **3** | **2** | **1** | **1** | **1** | **1** | **1** | **1** | **2** | **1** |
| **loci name** | **Genepriority score** | **Known HM genes** | **HM related genes** | **Ocular disease genes** | **Localized at MYP site** | **More than two mutation predict tools show damage** | **Exonic:protein alterning** | **Exonic:non-protein alterning** | **Expression in human adult ocular tissue** | **Expression in human embryonic ocular tissue** | **Frequency global-novel** | **Frequency global-rare** |
| ***CSMD1*** | **9** |  |  |  |  |  |  |  |  |  |  |  |
| ***FNDC3B*** | **8** |  |  |  |  |  |  |  |  |  |  |  |
| ***PARP8*** | **7** |  |  |  |  |  |  |  |  |  |  |  |
| ***ADAMTSL1*** | **7** |  |  |  |  |  |  |  |  |  |  |  |
| ***SGCA*** | **7** |  |  |  |  |  |  |  |  |  |  |  |
| ***ADAMTSL4*** | **7** |  |  |  |  |  |  |  |  |  |  |  |
| ***TENM4*** | **6** |  |  |  |  |  |  |  |  |  |  |  |
| ***VCAN*** | **6** |  |  |  |  |  |  |  |  |  |  |  |
| ***BBS1*** | **6** |  |  |  |  |  |  |  |  |  |  |  |
| ***SLC26A3*** | **6** |  |  |  |  |  |  |  |  |  |  |  |
| ***AMY2B*** | **6** |  |  |  |  |  |  |  |  |  |  |  |
| ***IDUA*** | **6** |  |  |  |  |  |  |  |  |  |  |  |
| ***TNC*** | **6** |  |  |  |  |  |  |  |  |  |  |  |
| ***COL16A1*** | **6** |  |  |  |  |  |  |  |  |  |  |  |
| ***ARHGEF6*** | **6** |  |  |  |  |  |  |  |  |  |  |  |
| ***CYP1B1*** | **6** |  |  |  |  |  |  |  |  |  |  |  |
| ***FOXL2*** | **6** |  |  |  |  |  |  |  |  |  |  |  |
| ***RPGR*** | **5** |  |  |  |  |  |  |  |  |  |  |  |
| ***TRANK1*** | **5** |  |  |  |  |  |  |  |  |  |  |  |
| ***FAAH2*** | **5** |  |  |  |  |  |  |  |  |  |  |  |
| ***COL28A1*** | **5** |  |  |  |  |  |  |  |  |  |  |  |
| ***TNIP1*** | **5** |  |  |  |  |  |  |  |  |  |  |  |
| ***DGKA*** | **5** |  |  |  |  |  |  |  |  |  |  |  |
| ***CFTR*** | **5** |  |  |  |  |  |  |  |  |  |  |  |
| ***MYH4*** | **5** |  |  |  |  |  |  |  |  |  |  |  |
| ***PIWIL2*** | **5** |  |  |  |  |  |  |  |  |  |  |  |
| ***ZNF157*** | **5** |  |  |  |  |  |  |  |  |  |  |  |
| ***MAPK1*** | **5** |  |  |  |  |  |  |  |  |  |  |  |
| ***ATP7A*** | **5** |  |  |  |  |  |  |  |  |  |  |  |
| ***PLCL1*** | **5** |  |  |  |  |  |  |  |  |  |  |  |
| ***DUOX2*** | **5** |  |  |  |  |  |  |  |  |  |  |  |
| ***PTGIS*** | **5** |  |  |  |  |  |  |  |  |  |  |  |
| ***AKR7A2*** | **5** |  |  |  |  |  |  |  |  |  |  |  |
| ***RGS18*** | **4** |  |  |  |  |  |  |  |  |  |  |  |
| ***CACNA1S*** | **4** |  |  |  |  |  |  |  |  |  |  |  |
| ***TYK2*** | **4** |  |  |  |  |  |  |  |  |  |  |  |
| ***ACE2*** | **4** |  |  |  |  |  |  |  |  |  |  |  |
| ***TMPRSS13*** | **4** |  |  |  |  |  |  |  |  |  |  |  |
| ***GARNL3*** | **4** |  |  |  |  |  |  |  |  |  |  |  |
| ***LARP1*** | **4** |  |  |  |  |  |  |  |  |  |  |  |
| ***BMPR2*** | **4** |  |  |  |  |  |  |  |  |  |  |  |
| ***DRP2*** | **4** |  |  |  |  |  |  |  |  |  |  |  |
| ***MUC16*** | **4** |  |  |  |  |  |  |  |  |  |  |  |
| ***TULP4*** | **4** |  |  |  |  |  |  |  |  |  |  |  |
| ***PAMR1*** | **4** |  |  |  |  |  |  |  |  |  |  |  |
| ***RDH16*** | **4** |  |  |  |  |  |  |  |  |  |  |  |
| ***ARSK*** | **4** |  |  |  |  |  |  |  |  |  |  |  |
| ***LAMC3*** | **4** |  |  |  |  |  |  |  |  |  |  |  |
| ***GPT*** | **4** |  |  |  |  |  |  |  |  |  |  |  |
| ***GALNS*** | **4** |  |  |  |  |  |  |  |  |  |  |  |
| ***SMARCAL1*** | **4** |  |  |  |  |  |  |  |  |  |  |  |
| ***MLH1*** | **4** |  |  |  |  |  |  |  |  |  |  |  |
| ***AK3*** | **4** |  |  |  |  |  |  |  |  |  |  |  |
| ***ISOC2*** | **4** |  |  |  |  |  |  |  |  |  |  |  |
| ***CRISP2*** | **3** |  |  |  |  |  |  |  |  |  |  |  |
| ***LRBA*** | **3** |  |  |  |  |  |  |  |  |  |  |  |
| ***MUT*** | **3** |  |  |  |  |  |  |  |  |  |  |  |
| ***BACH2*** | **3** |  |  |  |  |  |  |  |  |  |  |  |
| ***SPATA31C2*** | **2** |  |  |  |  |  |  |  |  |  |  |  |

**Supplementary Figure 2.** Genes ranked according to biological and statistical evidence. Genes were ranked based on 11 categories which can be divided in five categories: filter condition (green; genes are related to known HM genes, HM related genes , ocular disease genes or localized at MYP site), pathogenicity (light yellow; more than two mutation predict tools show damage ), annotation(light blue; genetic variant harboring an exonic protein altering variant or non-protein altering variant), expression (dark yellow; expression in adult human ocular tissue, expression in human embryonic ocular tissue), frequency (light dark; frequency global in ExAC database -novel, frequency global in ExAC database -rare).
