# Supplementary material for: Whole-Exome Sequencing in a Cohort of High Myopia Patients in Northwest China
Source: Front Cell Dev Biol. 2021 Jun 18;9:645501. doi: 10.3389/fcell.2021.645501 (PMC8250434; doi:10.3389/fcell.2021.645501)
Supplement: Supplementary file 1 [file Data_Sheet_1.zip › Supplemental Figure 3.DOCX]

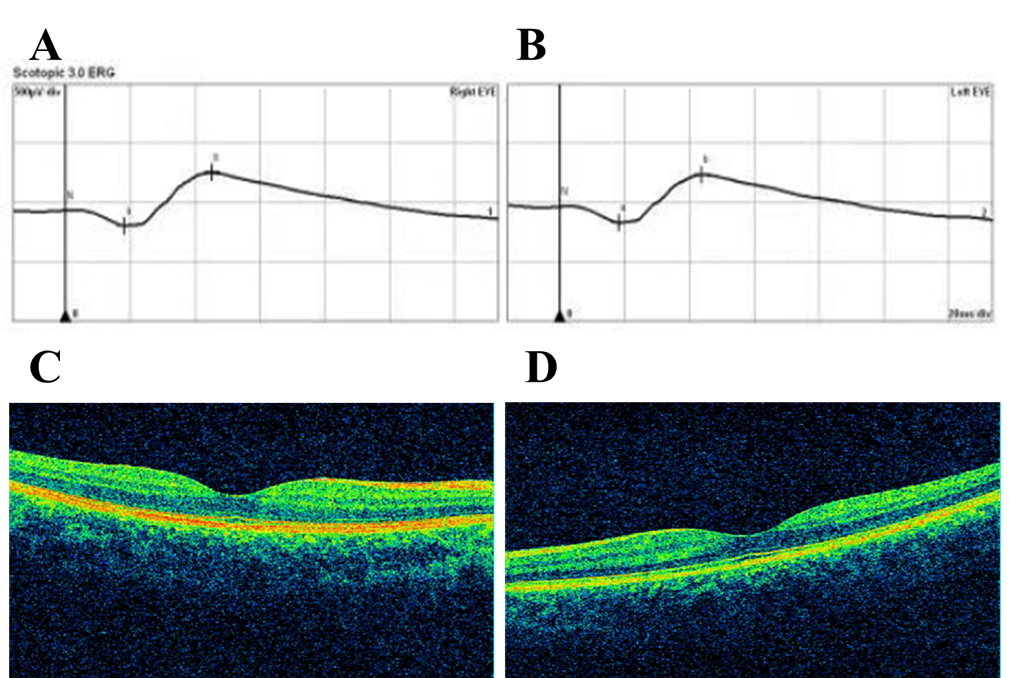


**Supplemental Figure 3.** OCT and ERG results of proband’s father in family 94. A, B: Standard scotopic response of full-field ERG of the right and left eye showing normal b-wave amplitude. C, D: OCT images of the right and left eye reveal a normal retinal appearance.
