# Supplementary material for: Whole-Exome Sequencing in a Cohort of High Myopia Patients in Northwest China
Source: Front Cell Dev Biol. 2021 Jun 18;9:645501. doi: 10.3389/fcell.2021.645501 (PMC8250434; doi:10.3389/fcell.2021.645501)
Supplement: Supplementary file 1 [file Data_Sheet_1.zip › Supplemental Figure 5.DOCX]

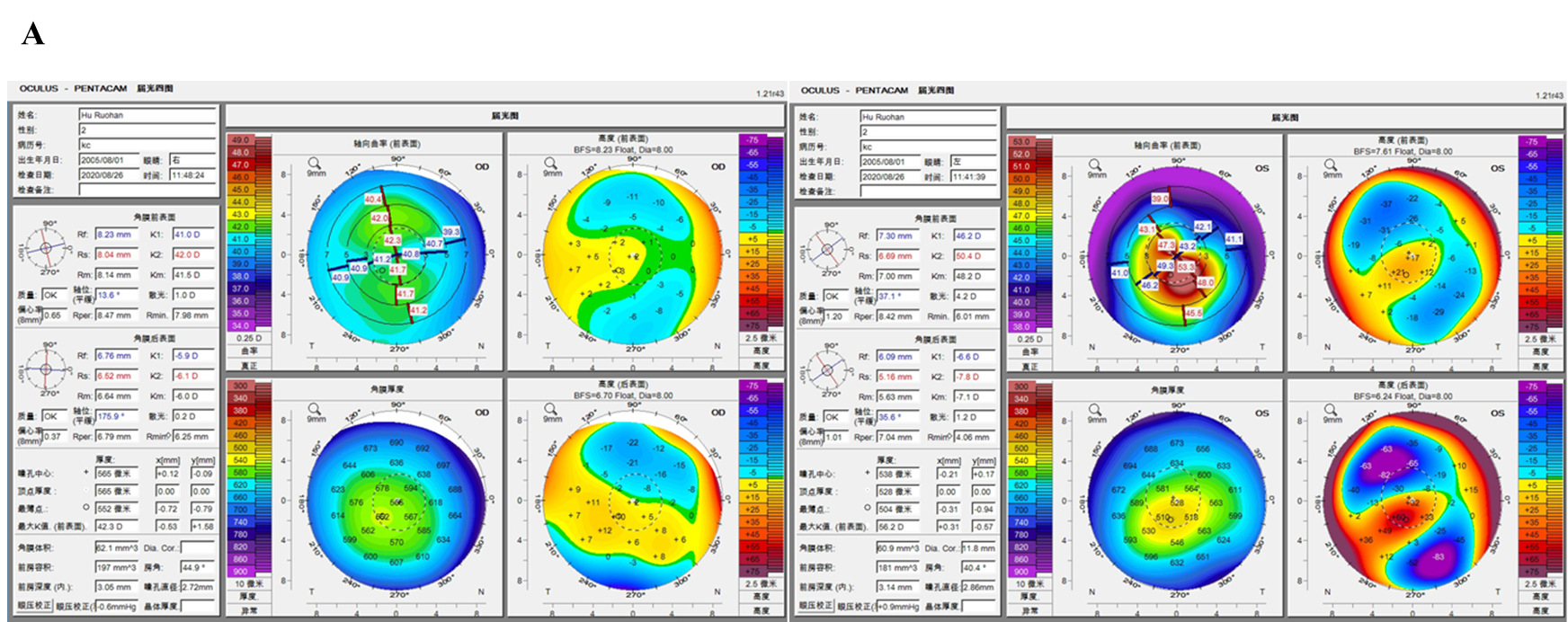


**Supplemental Figure 5:** The Scheimpflug 4 view display of the proband in family 104. From left to right, the images show the corneal conditions of the right and left eyes.

In each picture, the upper left and the lower left shows the curvature map and the thickness map; the upper right and lower right respectively shows the anterior elevation map and the posterior elevation map.
