# Supplementary material for: Whole-Exome Sequencing in a Cohort of High Myopia Patients in Northwest China
Source: Front Cell Dev Biol. 2021 Jun 18;9:645501. doi: 10.3389/fcell.2021.645501 (PMC8250434; doi:10.3389/fcell.2021.645501)
Supplement: Supplementary file 1 [file Data_Sheet_1.zip › Supplementary Table 1.DOCX]

**Supplemental Table 1. Complete gene list of genes**

**associated with myopia and ocular disease genes**

| **gene_symbol** | **Type** |
| --- | --- |
| ABCB7 | gene associated with myopia |
| ADAMTS18 | gene associated with myopia |
| ARR3 | gene associated with myopia |
| ATP8B1 | gene associated with myopia |
| BBS4 | gene associated with myopia |
| BSG | gene associated with myopia |
| C8orf37 | gene associated with myopia |
| CACNA1F | gene associated with myopia |
| CCDC111 | gene associated with myopia |
| CLDN16 | gene associated with myopia |
| COL11A1 | gene associated with myopia |
| COL18A1 | gene associated with myopia |
| COL2A1 | gene associated with myopia |
| COL9A2 | gene associated with myopia |
| CSMD1 | gene associated with myopia |
| CTSH | gene associated with myopia |
| CYP4V2 | gene associated with myopia |
| EPHB2 | gene associated with myopia |
| FAM161A | gene associated with myopia |
| FBN1 | gene associated with myopia |
| FOXP4 | gene associated with myopia |
| GLA | gene associated with myopia |
| GRM6 | gene associated with myopia |
| HIST1H3B | gene associated with myopia |
| LEPREL1 | gene associated with myopia |
| LRP2 | gene associated with myopia |
| LRPAP1 | gene associated with myopia |
| MAOA | gene associated with myopia |
| MYCBP2 | gene associated with myopia |
| MYP1 | gene associated with myopia |
| NYX | gene associated with myopia |
| OPN1LW | gene associated with myopia |
| OPN1MW | gene associated with myopia |
| OPTC | gene associated with myopia |
| P3H2 | gene associated with myopia |
| P4HA2 | gene associated with myopia |
| PAX6 | gene associated with myopia |
| PLXNA3 | gene associated with myopia |
| PPFIA2 | gene associated with myopia |
| PRDM5 | gene associated with myopia, keratoconus |
| PRIMPOL | gene associated with myopia |
| PROM1 | gene associated with myopia |
| PTPN11 | gene associated with myopia |
| PTPRR | gene associated with myopia |
| RP2 | gene associated with myopia |
| RPGR | gene associated with myopia |
| SCO2 | gene associated with myopia |
| SLC39A5 | gene associated with myopia |
| SLITRK6 | gene associated with myopia |
| STK11 | gene associated with myopia |
| TCF4 | gene associated with myopia |
| TENM4 | gene associated with myopia |
| TGFB1 | gene associated with myopia |
| TPSG1 | gene associated with myopia |
| UHRF1BP1L | gene associated with myopia |
| ZFHX1B | gene associated with myopia |
| ZNF469 | gene associated with myopia,keratoconus |
| ZNF644 | gene associated with myopia |
| A2BP1 | Ocular disease gene |
| ABCA1 | Ocular disease gene |
| ADAMTSL1 | Ocular disease gene |
| ANTXR2 | Ocular disease gene |
| ASPA | Ocular disease gene |
| B4GALNT2 | Ocular disease gene |
| BICC1 | Ocular disease gene |
| BLID | Ocular disease gene |
| BMP2 | Ocular disease gene |
| BMP3 | Ocular disease gene |
| BMP4 | Ocular disease gene |
| BMP6 | Ocular disease gene |
| CA8 | Ocular disease gene |
| CACNA1D | Ocular disease gene |
| CAPN9 | Ocular disease gene |
| CD55 | Ocular disease gene |
| CDH8 | Ocular disease gene |
| CHD4 | Ocular disease gene |
| CHD7 | Ocular disease gene |
| CHRNG | Ocular disease gene |
| CLNK | Ocular disease gene |
| CLSTN2 | Ocular disease gene |
| CNDP2 | Ocular disease gene |
| CNTN5 | Ocular disease gene |
| CTNND2 | Ocular disease gene |
| CYP26A1 | Ocular disease gene |
| DAOA-AS1 | Ocular disease gene |
| DENND1A | Ocular disease gene |
| DHX15 | Ocular disease gene |
| DLG2 | Ocular disease gene |
| DLX1 | Ocular disease gene |
| DNAH9 | Ocular disease gene |
| EHBP1L1 | Ocular disease gene |
| EPDR1 | Ocular disease gene |
| ERI1 | Ocular disease gene |
| FHIT | Ocular disease gene |
| FLI1 | Ocular disease gene |
| GATA4 | Ocular disease gene |
| GCSHP1 | Ocular disease gene |
| GJD2 | Ocular disease gene |
| GNPATP | Ocular disease gene |
| GOLGA8B | Ocular disease gene |
| GPR25 | Ocular disease gene |
| GRIA4 | Ocular disease gene |
| KCNJ2 | Ocular disease gene |
| KCNMA1 | Ocular disease gene |
| KCNQ5 | Ocular disease gene |
| KIAA1303 | Ocular disease gene |
| KNG1 | Ocular disease gene |
| LAMA2 | Ocular disease gene |
| LOC100506035 | Ocular disease gene |
| LRFN5 | Ocular disease gene |
| LRRC4C | Ocular disease gene |
| MIPEP | Ocular disease gene |
| MYO1D | Ocular disease gene |
| MYP11 | Ocular disease gene |
| NAV3 | Ocular disease gene |
| NPLOC4 | Ocular disease gene |
| NUDCD1 | Ocular disease gene |
| OR5M3 | Ocular disease gene |
| PABPCP2 | Ocular disease gene |
| PARP8 | Ocular disease gene |
| PCCA | Ocular disease gene |
| PCDH1 | Ocular disease gene |
| PDE11A | Ocular disease gene |
| PFKFB3 | Ocular disease gene |
| PGBD2 | Ocular disease gene |
| PML | Ocular disease gene |
| PPP1R3B | Ocular disease gene |
| PRSS56 | Ocular disease gene |
| PTPRD | Ocular disease gene |
| PTPRN2 | Ocular disease gene |
| PZP | Ocular disease gene |
| QKI | Ocular disease gene |
| RASGRF1 | Ocular disease gene |
| RBFOX1 | Ocular disease gene |
| RDH5 | Ocular disease gene |
| RFPL1 | Ocular disease gene |
| RGR | Ocular disease gene |
| RNA5SP144 | Ocular disease gene |
| RNU7-89P | Ocular disease gene |
| RORB | Ocular disease gene |
| ROS1 | Ocular disease gene |
| SAMD5 | Ocular disease gene |
| SCML4 | Ocular disease gene |
| SEMA4F | Ocular disease gene |
| SETMAR | Ocular disease gene |
| SFRP1 | Ocular disease gene |
| SH3GL2 | Ocular disease gene |
| SHISA6 | Ocular disease gene |
| SIX6 | Ocular disease gene |
| SNTB1 | Ocular disease gene |
| SPTBN1 | Ocular disease gene |
| SRPK2 | Ocular disease gene |
| TAC1 | Ocular disease gene |
| TACC2 | Ocular disease gene |
| TCF7L2 | Ocular disease gene |
| TJP2 | Ocular disease gene |
| TMEM261 | Ocular disease gene |
| TMEM98 | Ocular disease gene |
| TNIP1 | Ocular disease gene |
| TOX | Ocular disease gene |
| TPT1 | Ocular disease gene |
| UBE2HP1 | Ocular disease gene |
| VIPR2 | Ocular disease gene |
| WNT7B | Ocular disease gene |
| ZBTB38 | Ocular disease gene |
| ZC3H11B | Ocular disease gene |
| ZIC2 | Ocular disease gene |
| ZMAT4 | Ocular disease gene |
| ZNF285A | Ocular disease gene |
| ZNF536 | Ocular disease gene |
| ABCA4 | Ocular disease gene |
| ABCB6 | Ocular disease gene |
| ABCC6 | Ocular disease gene |
| ABHD12 | Ocular disease gene |
| ACBD5 | Ocular disease gene |
| ACO2 | Ocular disease gene |
| ADAM9 | Ocular disease gene |
| ADAMTS10 | Ocular disease gene |
| ADAMTS17 | Ocular disease gene |
| ADAMTSL4 | Ocular disease gene |
| ADGRA3 | Ocular disease gene |
| ADGRV1 | Ocular disease gene |
| ADIPOR1 | Ocular disease gene |
| AGBL1 | Ocular disease gene |
| AGBL5 | Ocular disease gene |
| AGK | Ocular disease gene |
| AHI1 | Ocular disease gene |
| AIPL1 | Ocular disease gene |
| ALDH1A3 | Ocular disease gene |
| ALMS1 | Ocular disease gene |
| ANGPTL3 | Ocular disease gene |
| ANOP1 | Ocular disease gene |
| AP3B1 | Ocular disease gene |
| AP3D1 | Ocular disease gene |
| APOA1 | Ocular disease gene |
| APOB | Ocular disease gene |
| APOE | Ocular disease gene |
| ARHGEF18 | Ocular disease gene |
| ARL13B | Ocular disease gene |
| ARL2BP | Ocular disease gene |
| ARL3 | Ocular disease gene |
| ARL6 | Ocular disease gene |
| ARMS2 | Ocular disease gene |
| ASB10 | Ocular disease gene |
| ASIP | Ocular disease gene |
| ASRGL1 | Ocular disease gene |
| ATF6 | Ocular disease gene |
| ATOH7 | Ocular disease gene |
| ATXN7 | Ocular disease gene |
| B3GLCT | Ocular disease gene |
| B4GAT1 | Ocular disease gene |
| B9D1 | Ocular disease gene |
| BBIP1 | Ocular disease gene |
| BBS1 | Ocular disease gene |
| BBS10 | Ocular disease gene |
| BBS12 | Ocular disease gene |
| BBS2 | Ocular disease gene |
| BBS5 | Ocular disease gene |
| BBS7 | Ocular disease gene |
| BBS9 | Ocular disease gene |
| BCOR | Ocular disease gene |
| BEST1 | Ocular disease gene |
| BFSP1 | Ocular disease gene |
| BFSP2 | Ocular disease gene |
| BLOC1S3 | Ocular disease gene |
| BLOC1S6 | Ocular disease gene |
| C10orf11 | Ocular disease gene |
| C12orf65 | Ocular disease gene |
| C1QTNF5 | Ocular disease gene |
| C2 | Ocular disease gene |
| C21orf2 | Ocular disease gene |
| C2orf71 | Ocular disease gene |
| C3 | Ocular disease gene |
| C5orf42 | Ocular disease gene |
| C9 | Ocular disease gene |
| CA4 | Ocular disease gene |
| CABP4 | Ocular disease gene |
| CACNA2D4 | Ocular disease gene |
| CAPN5 | Ocular disease gene |
| CC2D2A | Ocular disease gene |
| CCA1 | Ocular disease gene |
| CCDC28B | Ocular disease gene |
| CCT2 | Ocular disease gene |
| CCV | Ocular disease gene |
| CDH23 | Ocular disease gene |
| CDH3 | Ocular disease gene |
| CDHR1 | Ocular disease gene |
| CEP104 | Ocular disease gene |
| CEP164 | Ocular disease gene |
| CEP250 | Ocular disease gene |
| CEP290 | Ocular disease gene |
| CEP41 | Ocular disease gene |
| CEP78 | Ocular disease gene |
| CERKL | Ocular disease gene |
| CFB | Ocular disease gene |
| CFH | Ocular disease gene |
| CFHR1 | Ocular disease gene |
| CFHR3 | Ocular disease gene |
| CFI | Ocular disease gene |
| CHM | Ocular disease gene |
| CHMP4B | Ocular disease gene |
| CHN1 | Ocular disease gene |
| CHRDL1 | Ocular disease gene |
| CHST6 | Ocular disease gene |
| CIB2 | Ocular disease gene |
| CLDN19 | Ocular disease gene |
| CLN3 | Ocular disease gene |
| CLPB | Ocular disease gene |
| CLRN1 | Ocular disease gene |
| CLUAP1 | Ocular disease gene |
| CNA1 | Ocular disease gene |
| CNGA1 | Ocular disease gene |
| CNGA3 | Ocular disease gene |
| CNGB1 | Ocular disease gene |
| CNGB3 | Ocular disease gene |
| CNNM4 | Ocular disease gene |
| COL11A2 | Ocular disease gene |
| COL4A1 | Ocular disease gene,keratoconus |
| COL4A3 | Ocular disease gene,keratoconus |
| COL4A4 | Ocular disease gene,keratoconus |
| COL4A5 | Ocular disease gene |
| COL8A2 | Ocular disease gene |
| COL9A1 | Ocular disease gene |
| COLEC11 | Ocular disease gene |
| CORD1 | Ocular disease gene |
| CORD17 | Ocular disease gene |
| CORD8 | Ocular disease gene |
| CPAMD8 | Ocular disease gene |
| CRB1 | Ocular disease gene,keratoconus |
| CRX | Ocular disease gene,keratoconus |
| CRYAA | Ocular disease gene |
| CRYAB | Ocular disease gene |
| CRYBA1 | Ocular disease gene |
| CRYBA2 | Ocular disease gene |
| CRYBA4 | Ocular disease gene |
| CRYBB1 | Ocular disease gene |
| CRYBB2 | Ocular disease gene |
| CRYBB3 | Ocular disease gene |
| CRYGB | Ocular disease gene |
| CRYGC | Ocular disease gene |
| CRYGD | Ocular disease gene |
| CRYGS | Ocular disease gene |
| CSPP1 | Ocular disease gene |
| CST3 | Ocular disease gene |
| CTAA1 | Ocular disease gene |
| CTAA2 | Ocular disease gene |
| CTC1 | Ocular disease gene |
| CTDP1 | Ocular disease gene |
| CTNNA1 | Ocular disease gene |
| CTNS | Ocular disease gene |
| CTPL1 | Ocular disease gene |
| CX3CR1 | Ocular disease gene |
| CYMD | Ocular disease gene |
| CYP1B1 | Ocular disease gene |
| DAG1 | Ocular disease gene |
| DCN | Ocular disease gene |
| DGUOK | Ocular disease gene |
| DHDDS | Ocular disease gene |
| DHX38 | Ocular disease gene |
| DMD | Ocular disease gene |
| DNA2 | Ocular disease gene |
| DRAM2 | Ocular disease gene |
| DTHD1 | Ocular disease gene |
| DTNBP1 | Ocular disease gene |
| DURS1 | Ocular disease gene |
| EDN3 | Ocular disease gene |
| EDNRB | Ocular disease gene |
| EFEMP1 | Ocular disease gene |
| ELOVL4 | Ocular disease gene |
| ELP4 | Ocular disease gene |
| EMC1 | Ocular disease gene |
| EPHA2 | Ocular disease gene |
| ERCC6 | Ocular disease gene |
| ERCC8 | Ocular disease gene |
| EVR3 | Ocular disease gene |
| EXOSC2 | Ocular disease gene |
| EYA1 | Ocular disease gene |
| EYS | Ocular disease gene |
| FBLN5 | Ocular disease gene |
| FBN2 | Ocular disease gene |
| FGFR2 | Ocular disease gene |
| FKRP | Ocular disease gene |
| FKTN | Ocular disease gene |
| FLVCR1 | Ocular disease gene |
| FOXC1 | Ocular disease gene |
| FOXE3 | Ocular disease gene |
| FOXL2 | Ocular disease gene |
| FRMD7 | Ocular disease gene |
| FSCN2 | Ocular disease gene |
| FTL | Ocular disease gene |
| FYCO1 | Ocular disease gene |
| FZD4 | Ocular disease gene |
| GALK1 | Ocular disease gene |
| GCNT2 | Ocular disease gene |
| GDF3 | Ocular disease gene |
| GDF6 | Ocular disease gene |
| GFER | Ocular disease gene |
| GGCX | Ocular disease gene |
| GJA3 | Ocular disease gene |
| GJA8 | Ocular disease gene |
| GLC1B | Ocular disease gene |
| GLC1C | Ocular disease gene |
| GLC1D | Ocular disease gene |
| GLC1H | Ocular disease gene |
| GLC1I | Ocular disease gene |
| GLC1J | Ocular disease gene |
| GLC1K | Ocular disease gene |
| GLC1M | Ocular disease gene |
| GLC1N | Ocular disease gene |
| GLC3B | Ocular disease gene |
| GLC3C | Ocular disease gene |
| GMPPB | Ocular disease gene |
| GNAT1 | Ocular disease gene |
| GNAT2 | Ocular disease gene |
| GNB3 | Ocular disease gene |
| GNPTG | Ocular disease gene |
| GPR143 | Ocular disease gene |
| GPR179 | Ocular disease gene |
| GRK1 | Ocular disease gene |
| GUCA1A | Ocular disease gene |
| GUCA1B | Ocular disease gene |
| GUCY2D | Ocular disease gene |
| HARS | Ocular disease gene |
| HERC2 | Ocular disease gene |
| HGSNAT | Ocular disease gene |
| HK1 | Ocular disease gene |
| HMCN1 | Ocular disease gene |
| HMGB3 | Ocular disease gene |
| HMX1 | Ocular disease gene |
| HPS1 | Ocular disease gene |
| HPS3 | Ocular disease gene |
| HPS4 | Ocular disease gene |
| HPS5 | Ocular disease gene |
| HPS6 | Ocular disease gene |
| HSF4 | Ocular disease gene |
| HTRA1 | Ocular disease gene |
| IARS2 | Ocular disease gene |
| IDH3B | Ocular disease gene |
| IFT140 | Ocular disease gene |
| IFT172 | Ocular disease gene |
| IFT27 | Ocular disease gene |
| IFT74 | Ocular disease gene |
| IFT81 | Ocular disease gene |
| IGBP1 | Ocular disease gene |
| IMPDH1 | Ocular disease gene |
| IMPG1 | Ocular disease gene |
| IMPG2 | Ocular disease gene |
| INPP5E | Ocular disease gene |
| INVS | Ocular disease gene |
| IQCB1 | Ocular disease gene |
| IRF4 | Ocular disease gene |
| ISPD | Ocular disease gene |
| ITM2B | Ocular disease gene |
| JAG1 | Ocular disease gene |
| JAM3 | Ocular disease gene |
| KCNJ13 | Ocular disease gene |
| KCNV2 | Ocular disease gene |
| KERA | Ocular disease gene |
| KIAA0556 | Ocular disease gene |
| KIAA0586 | Ocular disease gene |
| KIAA1549 | Ocular disease gene |
| KIF11 | Ocular disease gene |
| KITLG | Ocular disease gene |
| KIZ | Ocular disease gene |
| KLHL7 | Ocular disease gene |
| KRT12 | Ocular disease gene |
| KRT3 | Ocular disease gene |
| LAMA1 | Ocular disease gene |
| LARGE1 | Ocular disease gene |
| LCA5 | Ocular disease gene |
| LCAT | Ocular disease gene |
| LEMD2 | Ocular disease gene |
| LIM2 | Ocular disease gene |
| LRAT | Ocular disease gene |
| LRIT3 | Ocular disease gene |
| LRP5 | Ocular disease gene |
| LSS | Ocular disease gene |
| LTBP2 | Ocular disease gene |
| LZTFL1 | Ocular disease gene |
| MAB21L2 | Ocular disease gene |
| MAF | Ocular disease gene |
| MAFB | Ocular disease gene |
| MAK | Ocular disease gene |
| MAPKAPK3 | Ocular disease gene |
| MASP1 | Ocular disease gene |
| MC1R | Ocular disease gene |
| MCDR3 | Ocular disease gene |
| MERTK | Ocular disease gene |
| MFN2 | Ocular disease gene |
| MFRP | Ocular disease gene |
| MFSD8 | Ocular disease gene |
| MIP | Ocular disease gene |
| MIR204 | Ocular disease gene |
| MITF | Ocular disease gene |
| MKKS | Ocular disease gene |
| MKS1 | Ocular disease gene |
| MT-ATP6 | Ocular disease gene |
| MT-TH | Ocular disease gene |
| MT-TL1 | Ocular disease gene |
| MTTP | Ocular disease gene |
| MT-TS2 | Ocular disease gene |
| MVK | Ocular disease gene |
| MYO7A | Ocular disease gene |
| MYOC | Ocular disease gene |
| MYP10 | Ocular disease gene |
| MYP12 | Ocular disease gene |
| MYP13 | Ocular disease gene |
| MYP14 | Ocular disease gene |
| MYP15 | Ocular disease gene |
| MYP16 | Ocular disease gene |
| MYP17 | Ocular disease gene |
| MYP18 | Ocular disease gene |
| MYP2 | Ocular disease gene |
| MYP3 | Ocular disease gene |
| MYP5 | Ocular disease gene |
| MYP7 | Ocular disease gene |
| MYP8 | Ocular disease gene |
| MYP9 | Ocular disease gene |
| NAA10 | Ocular disease gene |
| NBAS | Ocular disease gene |
| NDP | Ocular disease gene |
| NEK2 | Ocular disease gene |
| NEUROD1 | Ocular disease gene |
| NHS | Ocular disease gene |
| NMNAT1 | Ocular disease gene |
| NNO1 | Ocular disease gene |
| NPHP1 | Ocular disease gene |
| NPHP3 | Ocular disease gene |
| NPHP4 | Ocular disease gene |
| NR2E3 | Ocular disease gene |
| NR2F1 | Ocular disease gene |
| NRL | Ocular disease gene |
| NTF4 | Ocular disease gene |
| NYS2 | Ocular disease gene |
| NYS3 | Ocular disease gene |
| NYS4 | Ocular disease gene |
| OAT | Ocular disease gene |
| OCA2 | Ocular disease gene |
| OCA5 | Ocular disease gene |
| OFD1 | Ocular disease gene |
| OPA1 | Ocular disease gene |
| OPA2 | Ocular disease gene |
| OPA3 | Ocular disease gene |
| OPA4 | Ocular disease gene |
| OPA5 | Ocular disease gene |
| OPA6 | Ocular disease gene |
| OPA8 | Ocular disease gene |
| OPN1SW | Ocular disease gene |
| OPTN | Ocular disease gene |
| OTX2 | Ocular disease gene |
| OVOL2 | Ocular disease gene |
| PANK2 | Ocular disease gene |
| PAX2 | Ocular disease gene |
| PAX3 | Ocular disease gene |
| PCDH15 | Ocular disease gene |
| PCYT1A | Ocular disease gene |
| PDE6A | Ocular disease gene |
| PDE6B | Ocular disease gene |
| PDE6C | Ocular disease gene |
| PDE6D | Ocular disease gene |
| PDE6G | Ocular disease gene |
| PDE6H | Ocular disease gene |
| PDZD7 | Ocular disease gene |
| PEX1 | Ocular disease gene |
| PEX2 | Ocular disease gene |
| PEX7 | Ocular disease gene |
| PGK1 | Ocular disease gene |
| PHYH | Ocular disease gene |
| PIKFYVE | Ocular disease gene |
| PITPNM3 | Ocular disease gene |
| PITX2 | Ocular disease gene |
| PITX3 | Ocular disease gene |
| PLA2G5 | Ocular disease gene |
| PLK4 | Ocular disease gene |
| PNPLA6 | Ocular disease gene |
| POC1B | Ocular disease gene |
| POLG | Ocular disease gene |
| POLG2 | Ocular disease gene |
| POLR1C | Ocular disease gene |
| POLR1D | Ocular disease gene |
| POMGNT1 | Ocular disease gene |
| POMK | Ocular disease gene |
| POMT1 | Ocular disease gene |
| POMT2 | Ocular disease gene |
| PRCD | Ocular disease gene |
| PRDM13 | Ocular disease gene |
| PRPF3 | Ocular disease gene |
| PRPF31 | Ocular disease gene |
| PRPF4 | Ocular disease gene |
| PRPF6 | Ocular disease gene |
| PRPF8 | Ocular disease gene |
| PRPH2 | Ocular disease gene |
| PRPS1 | Ocular disease gene |
| PXDN | Ocular disease gene |
| RAB28 | Ocular disease gene |
| RARB | Ocular disease gene |
| RAX | Ocular disease gene |
| RAX2 | Ocular disease gene |
| RB1 | Ocular disease gene |
| RBP3 | Ocular disease gene |
| RBP4 | Ocular disease gene |
| RCBTB1 | Ocular disease gene |
| RD3 | Ocular disease gene |
| RDH11 | Ocular disease gene |
| RDH12 | Ocular disease gene |
| REEP6 | Ocular disease gene |
| RGS9 | Ocular disease gene |
| RGS9BP | Ocular disease gene |
| RHO | Ocular disease gene |
| RIMS1 | Ocular disease gene |
| RLBP1 | Ocular disease gene |
| RNASEH1 | Ocular disease gene |
| ROM1 | Ocular disease gene |
| RP1 | Ocular disease gene |
| RP1L1 | Ocular disease gene |
| RP22 | Ocular disease gene |
| RP24 | Ocular disease gene |
| RP29 | Ocular disease gene |
| RP32 | Ocular disease gene |
| RP34 | Ocular disease gene |
| RP6 | Ocular disease gene |
| RP63 | Ocular disease gene |
| RP9 | Ocular disease gene |
| RPE65 | Ocular disease gene |
| RPGRIP1 | Ocular disease gene |
| RPGRIP1L | Ocular disease gene |
| RRM2B | Ocular disease gene |
| RS1 | Ocular disease gene |
| RTN4IP1 | Ocular disease gene |
| RYR1 | Ocular disease gene |
| SAG | Ocular disease gene |
| SALL4 | Ocular disease gene |
| SAMD11 | Ocular disease gene |
| SDCCAG8 | Ocular disease gene |
| SEMA3E | Ocular disease gene |
| SEMA4A | Ocular disease gene |
| SHH | Ocular disease gene |
| SIPA1L3 | Ocular disease gene |
| SLC16A12 | Ocular disease gene |
| SLC24A1 | Ocular disease gene |
| SLC24A4 | Ocular disease gene |
| SLC24A5 | Ocular disease gene |
| SLC25A4 | Ocular disease gene |
| SLC25A46 | Ocular disease gene |
| SLC33A1 | Ocular disease gene |
| SLC45A2 | Ocular disease gene |
| SLC4A11 | Ocular disease gene,keratoconus |
| SLC4A4 | Ocular disease gene |
| SLC7A14 | Ocular disease gene |
| SMOC1 | Ocular disease gene |
| SNAI2 | Ocular disease gene |
| SNRNP200 | Ocular disease gene |
| SORD | Ocular disease gene |
| SOX10 | Ocular disease gene |
| SOX2 | Ocular disease gene |
| SPATA7 | Ocular disease gene |
| SPP2 | Ocular disease gene |
| STRA6 | Ocular disease gene |
| TACSTD2 | Ocular disease gene |
| TCOF1 | Ocular disease gene |
| TCTN1 | Ocular disease gene |
| TCTN2 | Ocular disease gene |
| TCTN3 | Ocular disease gene |
| TDRD7 | Ocular disease gene |
| TEAD1 | Ocular disease gene |
| TEK | Ocular disease gene |
| TENM3 | Ocular disease gene |
| TGFBI | Ocular disease gene,keratoconus |
| TIMM8A | Ocular disease gene |
| TIMP3 | Ocular disease gene |
| TK2 | Ocular disease gene |
| TLR3 | Ocular disease gene |
| TLR4 | Ocular disease gene |
| TMEM126A | Ocular disease gene |
| TMEM138 | Ocular disease gene |
| TMEM216 | Ocular disease gene |
| TMEM231 | Ocular disease gene |
| TMEM237 | Ocular disease gene |
| TMEM5 | Ocular disease gene |
| TMEM67 | Ocular disease gene |
| TOPORS | Ocular disease gene |
| TPCN2 | Ocular disease gene |
| TRAF3IP1 | Ocular disease gene |
| TREX1 | Ocular disease gene |
| TRIM32 | Ocular disease gene |
| TRIM44 | Ocular disease gene |
| TRNT1 | Ocular disease gene |
| TRPM1 | Ocular disease gene |
| TSPAN12 | Ocular disease gene |
| TTC8 | Ocular disease gene |
| TTLL5 | Ocular disease gene |
| TTPA | Ocular disease gene |
| TUB | Ocular disease gene |
| TUBGCP4 | Ocular disease gene |
| TUBGCP6 | Ocular disease gene |
| TULP1 | Ocular disease gene |
| TWNK | Ocular disease gene |
| TYR | Ocular disease gene |
| TYRP1 | Ocular disease gene |
| UBIAD1 | Ocular disease gene |
| UNC119 | Ocular disease gene |
| UNC45B | Ocular disease gene |
| USH1C | Ocular disease gene |
| USH1E | Ocular disease gene |
| USH1G | Ocular disease gene |
| USH1H | Ocular disease gene |
| USH1K | Ocular disease gene |
| USH2A | Ocular disease gene |
| VAX1 | Ocular disease gene |
| VCAN | Ocular disease gene |
| VIM | Ocular disease gene |
| VPS13B | Ocular disease gene |
| VSX1 | Ocular disease gene, keratoconus |
| VSX2 | Ocular disease gene |
| WDPCP | Ocular disease gene |
| WDR19 | Ocular disease gene |
| WDR36 | Ocular disease gene |
| WFS1 | Ocular disease gene |
| WHRN | Ocular disease gene |
| WS2B | Ocular disease gene |
| XYLT1 | gene associated with myopia |
| XYLT2 | Ocular disease gene |
| YAP1 | Ocular disease gene |
| YME1L1 | Ocular disease gene |
| ZEB1 | Ocular disease gene,keratoconus |
| ZNF408 | Ocular disease gene |
| ZNF423 | Ocular disease gene |
| ZNF513 | Ocular disease gene |
| ADAMTS2 | Ocular disease gene |
| ALDH18A1 | Ocular disease gene |
| BTD | Ocular disease gene |
| COL1A1 | Ocular disease gene, keratoconus |
| FGFR3 | Ocular disease gene |
| HSPG2 | Ocular disease gene |
| IFIH1 | Ocular disease gene |
| IFT122 | Ocular disease gene |
| IFT43 | Ocular disease gene |
| IKBKAP | Ocular disease gene |
| NIPBL | Ocular disease gene |
| NOTCH2 | Ocular disease gene |
| NSD1 | Ocular disease gene |
| NT5C2 | Ocular disease gene |
| RECQL4 | Ocular disease gene |
| SMC3 | Ocular disease gene |
| TFAP2A | Ocular disease gene |
| TRAPPC11 | Ocular disease gene |
| UBE3B | Ocular disease gene |
| WDR35 | Ocular disease gene |
| ZSWIM6 | Ocular disease gene |
| MMPs | Ocular disease gene |
| AGRN | gene associated with myopia |
| PLOD1 | Ocular disease gene |
| HGF | Ocular disease gene, keratoconus |
| TGIF1 | Ocular disease gene |
| LUM | gene associated with myopia |
| LOC399959 | Ocular disease gene |
| TMED3 | Ocular disease gene |
| ACTC1 | Ocular disease gene |
| FGF | Ocular disease gene |
| LOXL3 | gene associated with myopia |
| IGF-1 | gene associated with myopia |
| TEX28 | Ocular disease gene |
| DXYS15 | Ocular disease gene |
| DXS1073 | Ocular disease gene |
| DNAH11 | Ocular disease gene |
| EME1 | Ocular disease gene |
| ETNPPL | Ocular disease gene |
| AGXT2L1 | Ocular disease gene |
| HOXA2 | Ocular disease gene |
| PTPRQ | Ocular disease gene |
| TMPO | Ocular disease gene |
| TMTC2 | Ocular disease gene |
| TOB1 | Ocular disease gene |
| UNC5D | Ocular disease gene |
| CAST | Ocular disease gene, keratoconus |
| COL5A1 | Ocular disease gene, keratoconus |
| COL6A1 | Ocular disease gene, keratoconus |
| COL8A1 | Ocular disease gene, keratoconus |
| DOCK9 | Ocular disease gene, keratoconus |
| FNDC3B | Ocular disease gene, keratoconus |
| FOXO1 | Ocular disease gene, keratoconus |
| IL1A | Ocular disease gene, keratoconus |
| IL1B | Ocular disease gene, keratoconus |
| IL1RN | Ocular disease gene, keratoconus |
| LOX | Ocular disease gene, keratoconus |
| MIR184 | Ocular disease gene, keratoconus |
| MPDZ | Ocular disease gene, keratoconus |
| MPDZ-NF1B | Ocular disease gene, keratoconus |
| RAB3GAP1 | Ocular disease gene, keratoconus |
| RXRA | Ocular disease gene, keratoconus |
| SOD1 | Ocular disease gene, keratoconus |
| SPARC | Ocular disease gene, keratoconus |
| WNT10A | Ocular disease gene, keratoconus |
| DZIP1 | gene associated with myopia |
| XYLT1 | gene associated with myopia |
| NDUFAF7 | gene associated with myopia |
| CPSF1 | gene associated with myopia |
| TNFRSF21 | gene associated with myopia |
