# Supplementary material for: Whole-Exome Sequencing in a Cohort of High Myopia Patients in Northwest China
Source: Front Cell Dev Biol. 2021 Jun 18;9:645501. doi: 10.3389/fcell.2021.645501 (PMC8250434; doi:10.3389/fcell.2021.645501)
Supplement: Supplementary file 1 [file Data_Sheet_1.zip › Supplementary Table 2.DOCX]

**Supplementary Table 2.** De novo variants identified in families with high myopia

| **Family** | **Location** | **Gene** | **Variant type** | **Transcript** | **Mutation** | **Conservation** | **SIFT** | **Polyphen2_HDIV** | **Polyphen2_HVAR** | **LRT** | **MutationTaster** | **MutationAssessor** | **FATHMM** | **RadialSVM** | **LR** | **loss-of-function score** | **missense depletion score** | **gnomAD_exome_ALL** |
| --- | --- | --- | --- | --- | --- | --- | --- | --- | --- | --- | --- | --- | --- | --- | --- | --- | --- | --- |
| 90 | 6p24.3 | *BMP6* | nonframeshift deletion | NM_001718 | c.335_337del/p.112_113del | N/A | N/A | N/A | N/A | N/A | N/A | N/A | N/A | N/A | N/A | 0.18 | 1.18 | N/A |
| 90 | 6p24.3 | *BMP6* | nonframeshift insertion | NM_001718 | c.337_338insAGC/p.Q113delinsQQ | N/A | N/A | N/A | N/A | N/A | N/A | N/A | N/A | N/A | N/A | 0.18 | 1.18 | 0.000004922 |
| 93 | 5p13.2 | *NIPBL* | synonymous SNV | NM_015384 | c.C5874T/p.S1958S | N/A | N/A | N/A | N/A | N/A | N/A | N/A | N/A | N/A | N/A | 0.01 | 0.59 | 0.1507 |
| 93 | 9q34.3 | *COL5A1* | nonsynonymous SNV | NM_000093 | c.C61T/p.P21S | 0.09 | T | B | B | U | N/A | N/A | D | T | T | 0.02 | 0.83 | 0.001117 |
| 94 | Xp11.4 | *RPGR* | nonsynonymous SNV | NM_001034853 | c.A2808T/p.E936D | 2.881 | T | B | B | N/A | P | L | T | T | T | 0.04 | 0.82 | 0.01026 |
| 95 | 1q31.3 | *CFHR1* | nonsynonymous SNV | NM_002113 | c.G523C/p.E175Q | 0.066 | T | B | B | N/A | P | L | T | T | T | 0.95 | 1.07 | 0.1128 |
| 95 | 1q31.3 | *CFHR1* | synonymous SNV | NM_002113 | c.A588G/p.T196T | N/A | N/A | N/A | N/A | N/A | N/A | N/A | N/A | N/A | N/A | 0.95 | 1.07 | 0.1584 |
| 96 | 13q32.3 | *ZIC2* | nonframeshift deletion | NM_007129 | c.690_692del/p.230_231del | N/A | N/A | N/A | N/A | N/A | N/A | N/A | N/A | N/A | N/A | 0 | 0.41 | N/A |
| 96 | 13q32.3 | *ZIC2* | nonframeshift insertion | NM_007129 | c.692_693insCCA/p.H231delinsHH | N/A | N/A | N/A | N/A | N/A | N/A | N/A | N/A | N/A | N/A | 0 | 0.41 | N/A |
| 97 | 18q23 | *CTDP1* | nonsynonymous SNV | NM_004715 | c.T181G/p.S61A | 1.395 | T | B | B | N/A | P | N/A | T | T | T | 0.33 | 0.88 | 0.6149 |
| 99 | 6p25.3 | *FOXC1* | nonframeshift deletion | NM_001453 | c.1338_1340del/p.446_447del | N/A | N/A | N/A | N/A | N/A | N/A | N/A | N/A | N/A | N/A | 0 | 0.94 | N/A |
| 99 | 6p25.3 | *FOXC1* | nonframeshift insertion | NM_001453 | c.1340_1341insCGG/p.G447delinsGG | N/A | N/A | N/A | N/A | N/A | N/A | N/A | N/A | N/A | N/A | 0 | 0.94 | N/A |
| 101 | 16p12.3 | *XYLT1* | nonframeshift deletion | NM_022166 | c.268_270del/p.90_90del | N/A | N/A | N/A | N/A | N/A | N/A | N/A | N/A | N/A | N/A | 0.18 | 0.93 | N/A |
| 106 | 5q35.3 | *GRM6* | nonsynonymous SNV | NM_000843 | c.A176C/p.Q59P | 0.125 | T | B | B | N/A | P | N/A | T | T | T | 0.85 | 1.06 | 0.5498 |
| 111 | 18q23 | *CTDP1* | nonframeshift deletion | NM_001202504 | c.1371_1373del/p.457_458del | N/A | N/A | N/A | N/A | N/A | N/A | N/A | N/A | N/A | N/A | 0.33 | 0.88 | N/A |
| 111 | 18q23 | *CTDP1* | nonframeshift insertion | NM_001202504 | c.1373_1374insGGA/p.E458delinsEE | N/A | N/A | N/A | N/A | N/A | N/A | N/A | N/A | N/A | N/A | 0.33 | 0.88 | N/A |
| 112 | 4p15.2 | *ADGRA3* | synonymous SNV | NM_145290 | c.G693A/p.E231E | N/A | N/A | N/A | N/A | N/A | N/A | N/A | N/A | N/A | N/A | 0.23 | 0.95 | N/A |
| 112 | Xp22.2-p22.1 | *NHS* | nonsynonymous SNV | NM_001291867 | c.A47G/p.Q16R | 4.144 | D | P | B | N/A | D | N/A | T | T | T | 0 | 0.79 | N/A |
| 112 | Xp22.2-p22.1 | *NHS* | frameshift deletion | NM_001291867 | c.38_39del | N/A | N/A | N/A | N/A | N/A | N/A | N/A | N/A | N/A | N/A | 0 | 0.79 | N/A |
| 112 | Xp22.2-p22.1 | *NHS* | frameshift insertion | NM_001291867 | c.43_44insAG/p.R15fs | N/A | N/A | N/A | N/A | N/A | N/A | N/A | N/A | N/A | N/A | 0 | 0.79 | N/A |
| 116 | 18q23 | *CTDP1* | nonframeshift deletion | NM_001202504 | c.1371_1373del/p.457_458del | N/A | N/A | N/A | N/A | N/A | N/A | N/A | N/A | N/A | N/A | 0.33 | 0.88 | N/A |
| 116 | 18q23 | *CTDP1* | nonframeshift insertion | NM_001202504 | c.1373_1374insGGA/p.E458delinsEE | N/A | N/A | N/A | N/A | N/A | N/A | N/A | N/A | N/A | N/A | 0.33 | 0.88 | N/A |
