# Supplementary material for: Whole-Exome Sequencing in a Cohort of High Myopia Patients in Northwest China
Source: Front Cell Dev Biol. 2021 Jun 18;9:645501. doi: 10.3389/fcell.2021.645501 (PMC8250434; doi:10.3389/fcell.2021.645501)
Supplement: Supplementary file 1 [file Data_Sheet_1.zip › Supplementary Table 3.DOCX]

**Supplementary Table 3.** novel variants identified in families with high myopia

| **Family** | **Location** | **Gene** | **Variant type** | **Transcript** | **Mutation** | **Conservation** | **SIFT** | **Polyphen2_HDIV** | **Polyphen2_HVAR** | **LRT** | **MutationTaster** | **MutationAssessor** | **FATHMM** | **RadialSVM** | **LR** | **DANN_score** | **loss-of-function score** | **missense depletion score** | **gnomAD_exome_ALL** |
| --- | --- | --- | --- | --- | --- | --- | --- | --- | --- | --- | --- | --- | --- | --- | --- | --- | --- | --- | --- |
| 113 | 9p22.2-p22.1 | *ADAMTSL1* | nonsynonymous SNV | NM_001040272 | c.G848A/p.G283D | 4.761 | D | P | P | N/A | D | L | T | T | T | 0.98 | 0.4 | 1.08 | N/A |
| 104 | 1p21.1 | *AMY2B* | nonsynonymous SNV | NM_020978 | c.C655A/p.P219T | 7.686 | D | D | D | D | D | M | D | D | D | 0.997 | 1.33 | 1.43 | N/A |
| 113 | 19q13.32 | *APOC2* | frameshift deletion | NM_000483 | c.26delT/p.L9fs | N/A | N/A | N/A | N/A | N/A | N/A | N/A | N/A | N/A | N/A | N/A | 0.2 | 0.87 | N/A |
| 96 | Xq26.3 | *ARHGEF6* | nonsynonymous SNV | NM_004840 | c.G1068A/p.R625fs | 5.671 | D | D | D | D | D | L | T | T | T | 0.998 | 0.03 | 0.77 | N/A |
| 104 | Xq21.1 | *ATP7A* | nonsynonymous SNV | NM_000052 | c.G1154A/p.G385D | 9.869 | D | D | D | D | D | H | D | D | D | 0.998 | 0.09 | 0.82 | 0.0000056 |
| 106 | 16p13.3 | *C16orf71* | frameshift insertion | NM_139170 | c.435_436insTGGC /p.R145fs | N/A | N/A | N/A | N/A | N/A | N/A | N/A | N/A | N/A | N/A | N/A | 1.11 | 1.23 | N/A |
| 104 | 16p13.3 | *C16orf96* | frameshift deletion | NM_001145011 | c.2170delC/p.P724fs | N/A | N/A | N/A | N/A | N/A | N/A | N/A | N/A | N/A | N/A | N/A | 0.81 | 0.92 | N/A |
| 106 | 3p21.31 | *CAMP* | frameshift deletion | NM_004345 | c.195delC/p.D65fs | N/A | N/A | N/A | N/A | N/A | N/A | N/A | N/A | N/A | N/A | N/A | 0.7 | 0.95 | N/A |
| 104 | 16q12.2 | *CAPNS2* | frameshift deletion | NM_032330 | c.665delC/p.A222fs | N/A | N/A | N/A | N/A | N/A | N/A | N/A | N/A | N/A | N/A | N/A | 0.65 | 0.98 | N/A |
| 85 | 11q13.2 | *CARNS1* | frameshift insertion | NM_020811 | c.863_864insGCGGCAGC/p.V288fs | N/A | N/A | N/A | N/A | N/A | N/A | N/A | N/A | N/A | N/A | N/A | 0.54 | 0.87 | N/A |
| 96 | 7q31.2 | *CFTR* | nonsynonymous SNV | NM_000492 | c.G1392T/p.K464N | 5.92 | D | D | D | D | D | H | D | D | D | 0.999 | 1.09 | 1.32 | N/A |
| 93 | 1p35.2 | *COL16A1* | nonsynonymous SNV | NM_001856 | c.G2426A/p.G809E | 5.135 | D | D | D | D | D | H | D | D | D | 0.994 | 0.51 | 0.9 | N/A |
| 113 | 7p21.3 | *COL28A1* | nonsynonymous SNV | NM_001037763 | c.G1696C/p.G566R | 2.096 | D | D | D | U | D | H | D | D | D | 0.999 | 0.83 | 1.1 | N/A |
| 94 | 8p23.2 | *CSMD1* | nonsynonymous SNV | NM_033225 | c.C904T/p.R302C | 2.478 | T | D | D | N/A | D | M | D | D | D | 0.999 | 0.15 | 1.44 | N/A |
| 97 | 2p22.2 | *CYP1B1* | nonsynonymous SNV | NM_000104 | c.T284C/p.V95A | 4.849 | D | D | D | D | D | H | D | D | D | 0.994 | 0.67 | 1.12 | 0 |
| 104 | 3p14.1 | *EOGT* | frameshift deletion | NM_173654 | c.890delA/p.N297fs | N/A | N/A | N/A | N/A | N/A | N/A | N/A | N/A | N/A | N/A | N/A | 0.66 | 1..02 | N/A |
| 115 | 17p11.2 | *EPN2* | frameshift deletion | NM_001102664 | c.794_795del/p.P265fs | N/A | N/A | N/A | N/A | N/A | N/A | N/A | N/A | N/A | N/A | N/A | 0.19 | 0.8 | 0.00000924 |
| 96 | Xp11.21 | *FAAH2* | nonsynonymous SNV | NM_174912 | c.C364T/p./p122S | 2.12 | D | D | D | U | D | M | T | T | D | 0.998 | 1.59 | 1.75 | 0.00000569 |
| 113 | 12q13.13 | *FIGNL2* | nonsynonymous SNV | NM_001013690 | c.C1114A/p.P372T | N/A | N/A | N/A | N/A | N/A | N/A | N/A | N/A | N/A | N/A | N/A | N/A | N/A | 0.00000888 |
| 104 | 3q26.31 | *FNDC3B* | nonsynonymous SNV | NM_001135095 | c.A686G/p.H229R | 0 | D | P | P | D | D | M | T | T | T | 0.915 | 0 | 0.8 | 0 |
| 115 | 3p12.3 | *FRG2C* | frameshift deletion | NM_001124759 | c.129_130del/p.K43fs | N/A | N/A | N/A | N/A | N/A | N/A | N/A | N/A | N/A | N/A | N/A | N/A | N/A | N/A |
| 101 | Xp21.1 | *FTH1P18* | stopgain | NM_001271682 | c.C134A/p.S45X | N/A | N/A | N/A | N/A | N/A | N/A | N/A | N/A | N/A | N/A | N/A | N/A | N/A | N/A |
| 93 | 20p13 | *GNRH2* | frameshift insertion | NM_001501 | c.118-119insCAGCC /p.S40fs | N/A | N/A | N/A | N/A | N/A | N/A | N/A | N/A | N/A | N/A | N/A | 1.42 | 1.13 | N/A |
| 104 | 4p16.3 | *IDUA* | nonsynonymous SNV | NM_000203 | c.A770G/p.D257G | 7.236 | D | D | D | D | D | M | D | D | D | 0.998 | 0.82 | 1.09 | 0.00000993 |
| 94 | 19q13.33 | *KCNC3* | frameshift deletion | NM_004977 | c.1873_1874del/p.R625fs | N/A | N/A | N/A | N/A | N/A | N/A | N/A | N/A | N/A | N/A | N/A | 0.26 | 0.57 | N/A |
| 93 | 19q13.42 | *KIR3DL1* | frameshift deletion | NM_013289 | c.1313delC/p.S438fs | N/A | N/A | N/A | N/A | N/A | N/A | N/A | N/A | N/A | N/A | N/A | 1.24 | 1.3 | N/A |
| 115 | 12 | *LOC100129940* | frameshift insertion | NM_001292023 | c.248dupA/p.E83fs | N/A | N/A | N/A | N/A | N/A | N/A | N/A | N/A | N/A | N/A | N/A | N/A | N/A | 0.0000528 |
| 104 | 17q25.1 | *LOC100134391* | nonsynonymous SNV | NM_001278587 | c.G382A/p.G128S | N/A | N/A | N/A | N/A | N/A | N/A | N/A | N/A | N/A | N/A | N/A | N/A | N/A | N/A |
| 113 | 9q34.3 | *MAMDC4* | frameshift deletion | NM_206920 | c.2234_2249del/p.P745fs | N/A | N/A | N/A | N/A | N/A | N/A | N/A | N/A | N/A | N/A | N/A | 1.09 | 1.09 | N/A |
| 101 | 22q11.22 | *MAPK1* | nonsynonymous SNV | NM_002745 | c.A220T/p.I74F | 7.825 | D | D | D | D | D | L | T | T | T | 0.989 | 0 | 0.26 | N/A |
| 104 | 13q12.11 | *MICU2* | frameshift insertion | NM_152726 | c.1091dupA/p.H185R | N/A | N/A | N/A | N/A | N/A | N/A | N/A | N/A | N/A | N/A | N/A | 1.11 | 1.06 | N/A |
| 97 | 17p13.1 | *MYH4* | nonsynonymous SNV | NM_017533 | c.G547C/p.A183P | 7.739 | D | D | D | U | D | H | D | D | D | 0.997 | 0.88 | 0.99 | N/A |
| 97 | 12q13.3 | *PAN2* | frameshift deletion | NM_001127460 | c.3606_3607del/p.L1198fs | N/A | N/A | N/A | N/A | N/A | N/A | N/A | N/A | N/A | N/A | N/A | 0.19 | 0.66 | N/A |
| 97 | 11p15.5 | *PANO1* | nonsynonymous SNV | NM_001293167 | c.G238A/p.A80T | N/A | N/A | N/A | N/A | N/A | N/A | N/A | N/A | N/A | N/A | 0.91 | N/A | N/A | N/A |
| 104 | 20p11.22 | *PAX1* | frameshift deletion | NM_006192 | c.1364delG/p.H185R | N/A | N/A | N/A | N/A | N/A | N/A | N/A | N/A | N/A | N/A | N/A | 0.16 | 1.13 | N/A |
| 114 | 5q31.3 | *PCDHGA10* | frameshift insertion | NM_032090 | c.2467dupA/p.K823fs | N/A | N/A | N/A | N/A | N/A | N/A | N/A | N/A | N/A | N/A | N/A | 0.52 | 0.88 | N/A |
| 85 | 8p21.3 | *PIWIL2* | nonsynonymous SNV | NM_001135721 | c.G230T/p.G77V | 4.692 | D | D | D | D | D | L | T | T | T | 0.998 | 0.42 | 1.01 | N/A |
| 104 | 19p13.3 | *PLEKHJ1* | nonsynonymous SNV | NM_001300836 | c.A554G/p.H185R | N/A | N/A | N/A | N/A | N/A | N/A | N/A | N/A | N/A | N/A | 0.272 | 0.38 | 1.08 | 0 |
| 97 | 6q21 | *PRDM1* | frameshift deletion | NM_001198 | c.30delG/p.V10fs | N/A | N/A | N/A | N/A | N/A | N/A | N/A | N/A | N/A | N/A | N/A | 0.15 | 0.78 | N/A |
| 104 | 8q24.3 | *RECQL4* | nonsynonymous SNV | NM_004260 | c.G1100A/p.P418R | N/A | N/A | N/A | N/A | N/A | N/A | N/A | N/A | N/A | N/A | 0.766 | 0.96 | 1.43 | 0.00000817 |
| 93 | 17q23.1 | *RNFT1* | frameshift deletion | NM_016125 | c.1038_1039del/p.F346fs | N/A | N/A | N/A | N/A | N/A | N/A | N/A | N/A | N/A | N/A | N/A | 0.58 | 0.88 | N/A |
| 113 | 16p13.3 | *RNPS1* | frameshift insertion | NM_001286625 | c.26dupA/p.K9fs | N/A | N/A | N/A | N/A | N/A | N/A | N/A | N/A | N/A | N/A | N/A | 0 | 0.76 | N/A |
| 99 | Xp11.4 | *RPGR* | nonsynonymous SNV | NM_000328 | c.A2430C/p.V10fs | 1.513 | D | D | P | N/A | D | L | T | T | T | 0.997 | 0.04 | 0.82 | N/A |
| 93 | 17q21.33 | *SGCA* | nonsynonymous SNV | NM_001135697 | c.A629G/p.H210R | 8.348 | D | D | D | D | D | M | D | D | D | 0.995 | 0.42 | 0.88 | 0.00000435 |
| 94 | 22q13.1 | *SGSM3* | frameshift deletion | NM_001301849 | c.433_439del/p.R625fs | N/A | N/A | N/A | N/A | N/A | N/A | N/A | N/A | N/A | N/A | N/A | 0.66 | 0.98 | 0.0000041 |
| 91 | 20p13 | *SIGLEC1* | frameshift deletion | NM_023068 | c.4416delG/p.G1472fs | N/A | N/A | N/A | N/A | N/A | N/A | N/A | N/A | N/A | N/A | N/A | 1.01 | 1.02 | 0.00000815 |
| 97 | 7q22.3-q31.1 | *SLC26A3* | nonsynonymous SNV | NM_000111 | c.T1631A/p.I544N | 8.893 | D | D | D | D | D | M | D | D | D | 0.994 | 0.69 | 0.94 | N/A |
| 106 | 14q11.2 | *SLC7A8* | frameshift insertion | NM_001267037 | c.747dupC/p.K250fs | N/A | N/A | N/A | N/A | N/A | N/A | N/A | N/A | N/A | N/A | N/A | 0.65 | 0.97 | 0.00000406 |
| 94 | 14q32.33 | *TMEM179* | nonsynonymous SNV | NM_001286390 | c.G569A/p.R190H | N/A | N/A | N/A | N/A | N/A | N/A | N/A | N/A | N/A | N/A | 0.735 | 0 | 0.74 | N/A |
| 91 | 9q33.1 | *TNC* | nonsynonymous SNV | NM_002160 | c.T1129A/p.C377S | 9.279 | D | D | D | D | D | H | D | D | D | 0.971 | 0.33 | 1.01 | N/A |
| 91 | 3p22 | *TRANK1* | nonsynonymous SNV | NM_014831 | c.G4298A/p.R1433H | 6.023 | D | D | D | D | D | M | D | D | D | 1 | 0.54 | 0.85 | 0.00000407 |
| 113 | 11p15.5 | *TRPM5* | frameshift deletion | NM_014555 | c.539delT/p.L180fs | N/A | N/A | N/A | N/A | N/A | N/A | N/A | N/A | N/A | N/A | N/A | 0.77 | 0.99 | 0.00000816 |
| 94 | 5q14.2-q14.3 | *VCAN* | nonsynonymous SNV | NM_001164098 | c.G2601C/p.S40fs | -0.273 | D | P | B | N | N | L | T | T | T | 0.963 | 0.13 | 0.99 | N/A |
| 109 | Xp11.3 | *ZNF157* | nonsynonymous SNV | NM_003446 | c.T658C/p.C220R | 7.279 | D | D | D | N/A | D | H | D | D | D | 0.997 | 0.76 | 0.94 | N/A |
| 94 | 18p11.21 | *ZNF519* | frameshift insertion | NM_145287 | c.233dupA/p.G385D | N/A | N/A | N/A | N/A | N/A | N/A | N/A | N/A | N/A | N/A | N/A | 0.92 | 1.13 | N/A |
| 91 | 17p13.2 | *ZNF594* | frameshift deletion | NM_032530 | c.491_492del/p.S164fs | N/A | N/A | N/A | N/A | N/A | N/A | N/A | N/A | N/A | N/A | N/A | 1.01 | 1.35 | 0.0000163 |
| 104 | 4p16.3 | *ZNF718* | nonsynonymous SNV | NM_001039127 | c.C1349G/p.P418R | N/A | N/A | N/A | N/A | N/A | N/A | N/A | N/A | N/A | N/A | 0.627 | N/A | N/A | 0.00000416 |
| 110 | 16p11.2 | *ZNF747* | frameshift deletion | NM_001305018 | c.221delG/p.G74fs | N/A | N/A | N/A | N/A | N/A | N/A | N/A | N/A | N/A | N/A | N/A | 0.49 | 1.06 | N/A |
