# Supplementary material for: Whole-Exome Sequencing in a Cohort of High Myopia Patients in Northwest China
Source: Front Cell Dev Biol. 2021 Jun 18;9:645501. doi: 10.3389/fcell.2021.645501 (PMC8250434; doi:10.3389/fcell.2021.645501)
Supplement: Supplementary file 1 [file Data_Sheet_1.zip › Supplementary Table 4.DOCX]

**Supplementary Table 4**. rare variants identified in families with high myopia.

| **Family** | **Location** | **Gene** | **Variant type** | **Transcript** | **Mutation** | **Frequency in ExAC_ALL** | **Conservation** | **SIFT** | **Polyphen2_HDIV** | **Polyphen2_HVAR** | **LRT** | **MutationTaster** | **MutationAssessor** | **FATHMM** | **RadialSVM** | **LR** | **DANN_rankscore** | | **loss-of-function score** | **missense depletion score** | **gnomAD_exome_ALL** |
| --- | --- | --- | --- | --- | --- | --- | --- | --- | --- | --- | --- | --- | --- | --- | --- | --- | --- | --- | --- | --- | --- |
| 114 | Xp22.2 | *ACE2* | nonsynonymous SNV | NM_021804 | c.A1402G/p.I468V | 0.0007 | 6.28 | D | P | P | D | D | L | T | T | T | | 0.719 | 0.1 | 0.79 | 0.0008 |
| 97 | 10q26.3 | *ADAM8* | nonsynonymous SNV | NM_001164490 | c.G2142C/p.K714N | 0.00002616 | N/A | N/A | N/A | N/A | N/A | N/A | N/A | N/A | N/A | N/A | | 0.612 | 0.68 | 0.95 | 0.0000248 |
| 85 | 1q21.2 | *ADAMTSL4* | nonsynonymous SNV | NM_019032 | c.G2294A/p.R765H | 0.0001 | 6.132 | D | D | D | N/A | D | L | T | T | T | | 1 | 0.86 | 0.99 | 0.0002 |
| 91 | 6p12.3 | *ADGRF4* | frameshift deletion | NM_153838 | c.608delC/p.A203fs | 0.000008872 | N/A | N/A | N/A | N/A | N/A | N/A | N/A | N/A | N/A | N/A | | N/A | 0.86 | 1.08 | 0.0000131 |
| 113 | 9p24.1 | *AK3* | nonsynonymous SNV | NM_001199852 | c.C250T/p.R84C | 0.0000165 | 4.791 | D | D | D | D | D | H | D | D | D | | 0.999 | 0.74 | 1.5 | 0.00000813 |
| 97 | 1p36.13 | *AKR7A2* | nonsynonymous SNV | NM_003689 | c.G591C/p.Q197H | 0.000008249 | 4.365 | D | D | D | D | D | H | T | D | D | | 0.805 | 0.99 | 1.08 | 0.00000406 |
| 104 | 5q15 | *ARSK* | nonsynonymous SNV | NM_198150 | c.A214T/p.N72Y | 0.0007 | 7.097 | D | D | D | D | D | M | D | D | D | | 0.671 | 0.73 | 1.04 | 0.0006 |
| 93 | 11q13.2 | *BBS1* | nonsynonymous SNV | NM_024649 | c.G163A/p.V55M | 0.00009909 | 6.684 | D | P | P | N/A | D | M | D | D | D | | 0.939 | 0.69 | 0.98 | 0.0001 |
| 113 | 2q33.1-q33.2 | *BMPR2* | nonsynonymous SNV | NM_001204 | c.C1481T/p.A494V | 0.0000412 | 7.818 | D | D | D | D | D | M | D | D | D | | 0.973 | 0.08 | 0.76 | 0.0000609 |
| 97 | 10q22.2 | *C10orf55* | frameshift deletion | NM_001001791 | c.438delG/p.R146fs | 0.0001 | N/A | N/A | N/A | N/A | N/A | N/A | N/A | N/A | N/A | N/A | | N/A | 1.83 | 0.87 | 0.000091 |
| 113 | 1q32.1 | *CACNA1S* | nonsynonymous SNV | NM_000069 | c.C1465T/p.R489C | 0.0001 | 5.331 | D | D | P | D | D | M | D | D | D | | 0.992 | 0.39 | 1.01 | 0.0000609 |
| 85 | 3p14.3 | *CCDC66* | frameshift deletion | NM_001012506 | /c.963delA/p.S321fs | 0.0004 | N/A | N/A | N/A | N/A | N/A | N/A | N/A | N/A | N/A | N/A | | N/A | 0.97 | 1.14 | 0.0004 |
| 97 | 8q13.2 | *CPA6* | frameshift deletion | NM_020361 | c.54delC/p.C18fs | 0.00007448 | N/A | N/A | N/A | N/A | N/A | N/A | N/A | N/A | N/A | N/A | | N/A | 0.9 | 1.09 | 0.0001 |
| 85 | 6p12.3 | *CRISP2* | nonsynonymous SNV | NM_001142407 | c.T586C/p.C196R | 0.018 | 5.841 | D | D | D | D | D | M | T | T | T | | 0.823 | 1.14 | 1.11 | 0.0169 |
| 104 | 12q13.2 | *DGKA* | nonsynonymous SNV | NM_001345 | c.C2158T/p.L720F | 0.000008238 | 2.367 | D | D | D | D | D | M | D | D | D | | 0.959 | 0.22 | 0.65 | 0.0000204 |
| 109 | Xq22.1 | *DRP2* | nonsynonymous SNV | NM_001171184 | c.G1435A/p.E479K | 0.0000238 | 9.657 | D | D | D | D | D | M | T | D | D | | 0.952 | 0.41 | 0.76 | 0.0000116 |
| 104 | 15q21.1 | *DUOX2* | nonsynonymous SNV | NM_014080 | c.G3329A/p.R1110Q | 0.0002 | 7.713 | D | D | P | D | D | H | N/A | D | D | | 0.999 | 1.06 | 1.11 | 0.0002 |
| 104 | 8q24.3 | *EPPK1* | frameshift insertion | NM_031308 | c.108dupC/p.R37fs | 0.00007521 | N/A | N/A | N/A | N/A | N/A | N/A | N/A | N/A | N/A | N/A | | N/A | 0.96 | 1.23 | 0.0000823 |
| 113 | 1p13.2 | *FAM19A3* | frameshift deletion | NM_001004440 | c.307_311del/p.A103fs | 0.0002 | N/A | N/A | N/A | N/A | N/A | N/A | N/A | N/A | N/A | N/A | | N/A | 0.92 | 0.96 | 0.0002 |
| 113 | 21q22.3 | *FAM207A* | frameshift deletion | NM_058190 | c.260delG/p.R87fs | 0.0000828 | N/A | N/A | N/A | N/A | N/A | N/A | N/A | N/A | N/A | N/A | | N/A | 0.36 | 1.01 | 0.0000732 |
| 106 | 19q13.42 | *FCAR* | frameshift deletion | NM_133272 | c.170_171del/p.R57fs | 0.0002 | N/A | N/A | N/A | N/A | N/A | N/A | N/A | N/A | N/A | N/A | | N/A | 1 | 1.14 | 0.0003 |
| 93 | 1q21.3 | *FLG* | frameshift deletion | NM_002016 | c.3321delA/p.S1107fs | 0.0007 | N/A | N/A | N/A | N/A | N/A | N/A | N/A | N/A | N/A | N/A | | N/A | 2.42 | 2.15 | 0.0007 |
| 104 | 3q22.3 | *FOXL2* | nonsynonymous SNV | NM_023067 | c.C1045G/p.R349G | 0.0003 | 3.375 | D | D | D | N/A | D | L | D | D | D | | 0.568 | 0 | 0.62 | 0.0003 |
| 97 | 16q24.3 | *GALNS* | nonsynonymous SNV | NM_000512 | c.C857T/p.T286M | 0.0005 | 9.192 | D | D | D | D | D | M | D | D | D | | 0.919 | 0.69 | 1.07 | 0.0002 |
| 94 | 9q33.3 | *GARNL3* | nonsynonymous SNV | NM_032293 | c.A799T/p.T267S | 0.00001649 | 8.57 | N/A | D | D | D | D | M | D | D | D | | 0.759 | 0.35 | 0.76 | 0.00000813 |
| 93 | 13q12.11 | *GJB2* | frameshift deletion | NM_004004 | c.235delC/p.L79fs | 0.0004 | N/A | N/A | N/A | N/A | N/A | N/A | N/A | N/A | N/A | N/A | | N/A | 2.62 | 1.17 | 0.0004 |
| 104 | 9q34.11 | *GOLGA2* | frameshift deletion | NM_004486 | wholegene | 0.0002 | N/A | N/A | N/A | N/A | N/A | N/A | N/A | N/A | N/A | N/A | | N/A | 0.18 | 0.89 | 0.0001 |
| 91 | 2q31.1 | *GORASP2* | frameshift insertion | NM_001201428 | c.1042dupC/p.T347fs | 0.000008244 | N/A | N/A | N/A | N/A | N/A | N/A | N/A | N/A | N/A | N/A | | N/A | 0.15 | 0.71 | 0.00000813 |
| 106 | 8q24.3 | *GPT* | nonsynonymous SNV | NM_005309 | c.G968A/p.R323H | 0.00004516 | 6.251 | D | D | D | D | D | H | D | D | D | | 0.997 | 1.17 | 1.15 | 0.000065 |
| 113 | 6p22.2 | *HIST1H3D* | frameshift deletion | NM_003530 | c.281_291del/p.Q94fs | 0.0000494 | N/A | N/A | N/A | N/A | N/A | N/A | N/A | N/A | N/A | N/A | | N/A | 1.15 | 0.88 | 0.0000487 |
| 113 | 19q13.42 | *ISOC2* | nonsynonymous SNV | NM_001136201 | c.A65T/p.D22V | 0.0000907 | 7.026 | D | D | D | D | D | H | N/A | D | D | | 0.451 | 0.87 | 1.01 | 0.0000569 |
| 106 | 13q14.13 | *KIAA0226L* | frameshift insertion | NM_001286763 | c.21dupT/p.L8fs | 0.00009498 | N/A | N/A | N/A | N/A | N/A | N/A | N/A | N/A | N/A | N/A | | N/A | 0.93 | 0.96 | 0.0000551 |
| 113 | 9q32 | *KIF12* | frameshift deletion | NM_138424 | c.1486delC/p.Q496fs | 0.0000681 | N/A | N/A | N/A | N/A | N/A | N/A | N/A | N/A | N/A | N/A | | N/A | 0.68 | 0.89 | 0.0000784 |
| 94 | 12p13.31 | *KLRF2* | frameshift deletion | NM_001190765 | c.30_31del/p.L10fs | 0.00008165 | N/A | N/A | N/A | N/A | N/A | N/A | N/A | N/A | N/A | N/A | | N/A | 1.49 | 0.94 | 0.001 |
| 97 | 18q11.2 | ***LAMA3*** | nonsynonymous SNV | NM_001302996 | c.G1274A/p.R425H | 0.0002 | 7.812 | N/A | N/A | N/A | N/A | N/A | N/A | N/A | N/A | N/A | | 0.709 | 0.59 | 0.97 | 0.0002 |
| 104 | 9q34.12 | *LAMC3* | nonsynonymous SNV | NM_006059 | c.G449A/p.R150H | 0.000008291 | 9.835 | D | D | D | D | D | M | D | D | D | | 1 | 0.66 | 1.04 | 0.0000122 |
| 96 | 5q33.2 | *LARP1* | nonsynonymous SNV | NM_015315 | c.C2284T/p.R762C | 0.00002472 | 1.896 | D | D | D | D | D | M | T | T | T | | 0.986 | 0.07 | 0.65 | 0.0000284 |
| 115 | 6 | *LOC100996634* | nonsynonymous SNV | NM_001277339 | c.G764T/p.C255F | 0.0003 | N/A | N/A | N/A | N/A | N/A | N/A | N/A | N/A | N/A | N/A | | N/A | N/A | N/A | 0.0008 |
| 97 | 9 | *LOC286238* | nonsynonymous SNV | NM_001100111 | c.C17T/p.P6L | 0.00009559 | N/A | N/A | N/A | N/A | N/A | N/A | N/A | N/A | N/A | N/A | | N/A | N/A | N/A | 0.0000529 |
| 104 | 5q12.3 | *MAST4* | frameshift insertion | NM_001290227 | c.6791dupG | 0.0004 | N/A | N/A | N/A | N/A | N/A | N/A | N/A | N/A | N/A | N/A | | N/A | 0.28 | 0.86 | 0.0003 |
| 93 | 3p22.2 | *MLH1* | nonsynonymous SNV | NM_000249 | c.C649T/p.R217C | 0.0003 | 3.638 | D | D | D | D | D | H | D | D | D | | 0.998 | 0.37 | 1.04 | 0.0004 |
| 85 | 19p13.2 | *MUC16* | nonsynonymous SNV | NM_024690 | c.C41806T/p.R13936C | 0.0005 | 3.372 | N/A | D | D | N | N/A | M | T | T | T | | 0.959 | 0.68 | 1.27 | 0.0005 |
| 93 | 6p12.3 | *MUT* | nonsynonymous SNV | NM_000255 | c.A1286G/p.Y429C | 0.0001 | 3.278 | D | D | D | D | D | H | D | D | D | | 0.903 | N/A | N/A | 0.0001 |
| 85 | 6p21.1 | *NFKBIE* | frameshift deletion | NM_004556 | c.139delC/p.R47fs | 0.0006 | N/A | N/A | N/A | N/A | N/A | N/A | N/A | N/A | N/A | N/A | | N/A | 0.17 | 0.75 | 0.0004 |
| 97 | 1q42.13 | *OBSCN* | frameshift deletion | NM_001098623 | c.18775delA/p.R6259fs | 0.00008169 | N/A | N/A | N/A | N/A | N/A | N/A | N/A | N/A | N/A | N/A | | N/A | 0.79 | 1.04 | 0.0000731 |
| 85 | 1q44 | *OR2T35* | stopgain | NM_001001827 | c.957_958insTG/p.I320_R321delinsX | 0.0015 | N/A | N/A | N/A | N/A | N/A | N/A | N/A | N/A | N/A | N/A | | N/A | 0.39 | 1.12 | 0.0015 |
| 104 | 1q44 | *OR2W5* | nonsynonymous SNV | NM_001004698 | c.G673A/p.G225S | 0.0112 | N/A | N/A | N/A | N/A | N/A | N/A | N/A | N/A | N/A | N/A | | N/A | N/A | N/A | 0.0113 |
| 97 | 11p13 | *PAMR1* | nonsynonymous SNV | NM_001282676 | c.G1157A/p.R386H | 0.0003 | 4.922 | D | D | D | D | D | M | D | D | D | | 0.99 | 1.11 | 1.06 | 0.0002 |
| 85 | 5q11.1 | *PARP8* | nonsynonymous SNV | NM_001178056 | c.G860A/p.R287H | 0.0014 | 8.094 | N/A | D | D | D | D | N | N/A | T | T | | 0.998 | 0.25 | 0.73 | 0.0013 |
| 104 | 5q31 | *PCDHGB5* | nonsynonymous SNV | NM_018925 | c.C1726T/p.R576C | 0.0001 | N/A | N/A | N/A | N/A | N/A | N/A | N/A | N/A | N/A | N/A | | 0.204 | N/A | N/A | 0.0001 |
| 115 | 2q31.2 | *PDE11A* | frameshift deletion | NM_001077197 | c.20_21del/p.R7fs | 0.0029 | N/A | N/A | N/A | N/A | N/A | N/A | N/A | N/A | N/A | N/A | | N/A | 1.11 | 1 | 0.0031 |
| 115 | 4q22.3 | *PDHA2* | frameshift deletion | NM_005390 | c.245_246del/p.Q82fs | 0.0005 | N/A | N/A | N/A | N/A | N/A | N/A | N/A | N/A | N/A | N/A | | N/A | 1.15 | 1.05 | 0.0005 |
| 93 | 2q33.1 | *PLCL1* | nonsynonymous SNV | NM_006226 | c.G641T/p.W214L | 0.0003 | 9.8 | N/A | D | D | D | D | M | D | D | D | | 0.464 | 0.29 | 0.78 | 0.0003 |
| 106 | 16q22.2 | *PMFBP1* | frameshift deletion | NM_031293 | c.2882delG/p.G961fs | 0.0004 | N/A | N/A | N/A | N/A | N/A | N/A | N/A | N/A | N/A | N/A | | N/A | 0.88 | 1.06 | 0.0004 |
| 93 | 2q21.1 | *POTEE* | frameshift insertion | NM_001083538 | c.2049dupG/p.V683fs | 0.0006 | N/A | N/A | N/A | N/A | N/A | N/A | N/A | N/A | N/A | N/A | | N/A | 0.48 | 1.41 | 0.0003 |
| 97 | 2q37.3 | *PRR21* | frameshift deletion | NM_001080835 | c.264_319del/p.S88fs | 0.00003297 | N/A | N/A | N/A | N/A | N/A | N/A | N/A | N/A | N/A | N/A | | N/A | N/A | 0.87 | 0.0004 |
| 93 | 20q13.13 | *PTGIS* | nonsynonymous SNV | NM_000961 | c.G1339A/p.A447T | 0.0004 | 7.096 | D | D | D | D | D | M | D | D | D | | 0.84 | 0.73 | 1.06 | 0.0004 |
| 115 | 12q13.3 | *RDH16* | nonsynonymous SNV | NM_003708 | c.G227T/p.R76M | 0.0004 | 1.287 | D | D | D | D | D | M | D | D | D | | 0.288 | 0.95 | 1.02 | 0.0004 |
| 85 | 1q31.2 | *RGS18* | nonsynonymous SNV | NM_130782 | c.G352A/p.A118T | 0.0000168 | 6.349 | D | P | P | D | D | M | T | T | T | | 0.973 | 0.55 | 1.02 | 0.00000424 |
| 97 | 7q31.32 | *RNF148* | frameshift insertion | NM_198085 | c.582dupA/p.F195fs | 0.0004 | N/A | N/A | N/A | N/A | N/A | N/A | N/A | N/A | N/A | N/A | | N/A | 0.91 | 1.33 | 0.0004 |
| 104 | 6q22.31 | *RNF217* | nonsynonymous SNV | NM_001286398 | c.G451A/p.G151S | 0.0004 | N/A | N/A | N/A | N/A | N/A | N/A | N/A | N/A | N/A | N/A | | 0.38 | 0.59 | 0.69 | 0.0003 |
| 91 | 8p23.1 | *RP1L1* | frameshift insertion | NM_178857 | c.416dupC/p.P139fs | 0.0055 | N/A | N/A | N/A | N/A | N/A | N/A | N/A | N/A | N/A | N/A | | N/A | 1.72 | 1.77 | 0.0062 |
| 91 | 6q23.1 | *SAMD3* | frameshift deletion | NM_001277185 | c.258delA/p.K86fs | 0.000008243 | N/A | N/A | N/A | N/A | N/A | N/A | N/A | N/A | N/A | N/A | | N/A | 0.87 | 0.98 | 0.00000407 |
| 104 | 3p22.2 | *SCN10A* | frameshift insertion | NM_001293307 | c.4734dupC/p.Y1579fs | 0.000008243 | N/A | N/A | N/A | N/A | N/A | N/A | N/A | N/A | N/A | N/A | | N/A | 0.82 | 1.06 | 0.0000285 |
| 93 | 2q35 | *SMARCAL1* | nonsynonymous SNV | NM_001127207 | c.C1439T/p.P480L | 0.000008241 | 7.465 | D | D | D | D | D | H | D | D | D | | 0.985 | 0.41 | 0.89 | 0.00000812 |
| 115 | 9q22.1 | *SPATA31C1* | nonsynonymous SNV | NM_001145124 | c.G1808A/p.R603H | 0.0002 | N/A | N/A | N/A | N/A | N/A | N/A | N/A | N/A | N/A | N/A | | N/A | N/A | N/A | 0.0000978 |
| 113 | 9q22.1 | *SPATA31C2* | nonsynonymous SNV | NM_001166137 | c.G1979A/p.G660D | 0.0003 | N/A | N/A | N/A | N/A | N/A | N/A | N/A | N/A | N/A | N/A | | N/A | N/A | N/A | 0.0003 |
| 113 | 9q21.32 | *SPATA31D4* | nonsynonymous SNV | NM_001145197 | c.A2695T/p.M899L | 0.0002 | N/A | N/A | N/A | N/A | N/A | N/A | N/A | N/A | N/A | N/A | | N/A | N/A | N/A | 0.0000813 |
| 93 | 7q36.1 | *SSPO* | nonsynonymous SNV | NM_198455 | c.G6395A/p.R2132Q | 0.0002 | N/A | N/A | N/A | N/A | N/A | N/A | N/A | N/A | N/A | N/A | | 0.127 | N/A | N/A | 0.0000945 |
| 113 | 14q13 | *SSTR1* | stopgain | NM_001049 | c.1169_1170insGCTCTGAGCCCGGGCCACGCAGGG/p.T390delinsTLX | 0.0037 | N/A | N/A | N/A | N/A | N/A | N/A | N/A | N/A | N/A | N/A | | N/A | 0.11 | 0.78 | N/A |
| 91 | 11q23.3 | *TMPRSS13* | nonsynonymous SNV | NM_001206789 | c.A542C/p.D181A | 0.0002 | 5.165 | D | D | D | D | D | M | D | D | D | | 0.768 | 0.6 | 0.85 | 0.0002 |
| 113 | 5q33.1 | *TNIP1* | nonsynonymous SNV | NM_001252386 | c.C628T/p.R210W | 0.0023 | 1.238 | D | D | P | N | N | L | T | T | T | | 0.501 | 0.22 | 0.89 | 0.0024 |
| 91 | 6q25.3 | *TULP4* | nonsynonymous SNV | NM_020245 | c.G4396A/p.V1466M | 0.0003 | 6.144 | D | D | D | D | D | M | D | D | D | | 0.968 | 0.05 | 0.88 | 0.0003 |
| 113 | 19p13.2 | *TYK2* | nonsynonymous SNV | NM_003331 | c.G2102C/p.R701T | 0.0002 | 1.672 | D | D | D | N | D | M | D | D | D | | 0.354 | 0.4 | 0.84 | 0.0003 |
| 91 | 5p13.2 | *UGT3A1* | frameshift insertion | NM_152404 | c.216dupA/p.S73fs | 0.00002648 | N/A | N/A | N/A | N/A | N/A | N/A | N/A | N/A | N/A | N/A | | N/A | 1.01 | 1.02 | 0.0000164 |
| 104 | 3p21.31 | *XCR1* | frameshift insertion | NM_001024644 | c.874dupG/p.V292fs | 0.0003 | N/A | N/A | N/A | N/A | N/A | N/A | N/A | N/A | N/A | N/A | | N/A | 0.47 | 0.78 | 0.0002 |
| 93 | 7q11.21 | *ZNF107* | frameshift insertion | NM_001282360 | c.2447dupA/p.E816fs | 0.0073 | N/A | N/A | N/A | N/A | N/A | N/A | N/A | N/A | N/A | N/A | | N/A | 1.17 | 1.08 | 0.0029 |
| 104 | 19q13.42 | *ZNF600* | stopgain | NM_198457 | c.1326delC/p.Y442X | 0.0005 | N/A | N/A | N/A | N/A | N/A | N/A | N/A | N/A | N/A | N/A | | N/A | N/A | 1.26 | 0.0005 |
| 115 | 19p12 | *ZNF626* | frameshift deletion | NM_001076675 | c.746_747del/p.R249fs | 0.0007 | N/A | N/A | N/A | N/A | N/A | N/A | N/A | N/A | N/A | N/A | | N/A | 0.19 | 1.11 | 0.0007 |
| 113 | 4p16.3 | *ZNF721* | frameshift deletion | NM_133474 | c.1002_1003del/p.T334fs | 0.0000828 | N/A | N/A | N/A | N/A | N/A | N/A | N/A | N/A | N/A | N/A | | N/A | 0.93 | 1.23 | 0.000065 |
| 93 | 19q13.12 | *ZNF850* | nonsynonymous SNV | NM_001267779 | c.G1796A/p.R599Q | 0.00007822 | N/A | N/A | N/A | N/A | N/A | N/A | N/A | N/A | N/A | N/A | | 0.108 | 0 | 0.64 | 0.000052 |
